# Evolutionary game analysis of emergency medical supply production capacity reserves in nonprofit organizations: A theoretical perspective

**DOI:** 10.1371/journal.pone.0346435

**Published:** 2026-04-10

**Authors:** Hua Xiao, Guoqiang Luo, Yisheng Wang, Haijun Shuang, Bo Li

**Affiliations:** 1 PLA Joint Logistics Support Force University of Engineering, Shapingba, Chongqing, China; 2 Chongqing College of Humanities, Science & Technology, Hechuan, Chongqing, China; Zhejiang Gongshang University, CHINA

## Abstract

Since the 21st century, global public health emergencies have occurred frequently, posing severe threats to the economic development and social stability of countries worldwide. Against this backdrop, emergency medical supply reserves are particularly crucial—especially the production capacity reserves of non-durable medical materials characterized by high demand, short production cycles, and limited shelf lives. As a theoretical modeling study, this paper adopts the core ideas and analytical methods of evolutionary game theory to construct dynamic evolutionary game models for the cooperative production capacity reserves of emergency medical supplies between nonprofit organizations (NPOs) and reserves enterprises under two scenarios: “perfect competition” and “government incentive policy intervention.” Key factors including reputation benefits, social disaster reduction benefits, and opportunity costs are incorporated to systematically explore the impacts of multiple variables on the decision-making of both cooperative parties. Meanwhile, numerical simulation is employed to quantitatively analyze the operational mechanisms of government incentive policies, specifically one-time subsidies, revenue sharing, cost subsidies, and supervisory penalties. The research findings are as follows: First, the synergistic effect of perfect competition and government incentive policies can promote the evolutionary game system toward cooperative equilibrium. Among these factors, the impact of the incentive allocation coefficient is context-dependent, requiring scientific calibration to balance the interest demands of both parties. Second, the reserves cost-benefit ratio is a core threshold variable determining cooperative behavior. Perfect competition alone tends to fail under high cost-benefit ratios, while the “government-led—NPO-supplemented” collaborative incentive model can break through this threshold constraint via “long-term empowerment + short-term efficiency improvement.” Third, enterprises’ opportunity costs exert a significant negative impact on cooperation, whereas supervisory penalties exceeding a critical threshold can inhibit speculative behaviors and enhance cooperative willingness. Finally, the research findings of this paper are verified through simulation with the background of emergency medical reserves in Chongqing, China. Based on these findings, this study proposes countermeasures including optimizing the incentive allocation mechanism, constructing a collaborative incentive system, and improving the disciplinary constraint mechanism. This research provides new perspectives and practical guidance for optimizing the emergency medical material reserves system and ensuring the stable development of emergency reserves cooperation.

## 1 Introduction

Since the 21st century, public health emergencies have shown a high incidence globally, ranging from Severe Acute Respiratory Syndrome (SARS), Ebola virus disease, and Coronavirus Disease 2019 (COVID-19) to the monkeypox epidemic and cholera. Such public health crises have gradually evolved from sporadic incidents into regular challenges, posing a severe threat to the economic development and social stability of countries worldwide. Against this backdrop, emergency medical supply reserves—a systematic and planned stockpiling mechanism that involves the targeted collection, storage, and management of essential medical materials, equipment, and consumables to ensure their timely and effective deployment for emergency rescue, epidemic control, and medical treatment in the event of sudden public health incidents—form the core material support for emergency rescue [1–3]. Particularly during public health emergencies, the demand for key medical supplies such as reagents, protective clothing, and masks is extremely urgent. Due to their inherent characteristics of short production cycles and limited shelf lives, adopting capacity reserve models for targeted stockpiling has become a critical strategy in the construction of the emergency medical supply reserve system. [4–6].

Governments around the world have successively introduced emergency medical supplies reserves management measures, with the core objective of incentivizing medical supplies manufacturers to actively participate in reserves capacity building [7]. As important participants and direct implementers of the emergency medical supplies reserves system, enterprises’ proactive participation is crucial to the effective operation of the system. To fully mobilize enterprises’ enthusiasm for participating in reserves work, governments and Non-Profit Organizations (NPOs) have established a two-way regulatory mechanism of “incentives + constraints,” which specifically includes incentive measures such as one-time subsidies, social reserves revenue sharing, cost subsidies, and performance rewards, as well as restrictive measures such as regulatory penalties.

In the public health emergency response system, the stable supply of emergency medical supplies is a core prerequisite for ensuring rescue effectiveness. NPOs, relying on their social public welfare attributes and flexible resource allocation advantages, play an irreplaceable supporting role in this field. However, for NPOs, how to promote enterprises’ in-depth participation in emergency medical supplies capacity reserves through scientific and effective guidance mechanisms, fully exert the effectiveness of enterprises’ production capacity reserves, and thereby systematically improve the overall emergency rescue support capacity, has become a core proposition urgently needing to be addressed in the construction and high-quality development of the emergency medical supplies reserves system. As an important part of the community with a shared future for mankind, enterprises bear the social responsibility of responding to crises in the context of public health emergencies. Therefore, as collaborative agents in emergency reserves, NPOs can either adopt the entrusted reserves model by signing reserves agreements or opt out of entrusted reserves when cooperating with enterprises, and instead implement a market reserves strategy as a functional alternative to entrusted reserves.

Scholars have conducted in-depth research on emergency medical supplies reserves, but there are still two key research gaps: first, the role positioning and functional boundaries of NPOs in the context of government incentive policies have not been fully clarified; second, there is a lack of systematic theoretical connection with the foundational literature of Evolutionary Game Theory (EGT), which underpins the methodological framework of this study. As a core theoretical tool for analyzing dynamic multi-agent interactions, EGT originated from Smith’s pioneering work Evolution and the Theory of Games (1982), which laid the theoretical foundation for evolutionary stability and replicator dynamics [8]. Subsequent studies have further expanded this theoretical framework to heterogeneous agent scenarios—this is highly consistent with the unique attributes of NPOs and enterprises. A typical example is Samuelson’s Evolutionary Games and Equilibrium Selection (1997), which clarified the strategy selection mechanism in asymmetric agent contexts and provided important theoretical support for multi-agent interaction analysis [9]. In the field of demand forecasting and procurement, Zhang et al. focused on post-disaster material shortages and verified through an evolutionary game model that the achievement of multi-agent cooperation equilibrium among governments, enterprises, and relief organizations depends on the coordinated design of subsidy intensity, cost-sharing, and risk-sharing mechanisms, with their research implicitly drawing on the core ideas of the aforementioned EGT [4]. Other studies, through cross-country comparative analysis during the COVID-19 pandemic, identified key issues such as insufficient reserves scale and weak emergency mobilization capacity, thereby emphasizing the practical urgency of upgrading the capacity reserves system [5]; recent application practices of EGT in the medical supplies field have fully confirmed the unique value of this theory in addressing supply chain coordination challenges [10].

In research related to multi-party cooperation, academic achievements based on EGT have provided important methodological support and empirical references for this study. For instance, Baumann et al. proposed a computational method for evolutionary coordination games with heterogeneous agents, offering key technical support for the construction of multi-agent interaction models [11]; analyses of manufacturers’ capacity constraints and studies on production and ordering response strategies under uncertain demand in a two-tier supply chain composed of a social planner and a manufacturer have shown that the centralized supply chain management model can effectively reduce ordering costs—this is consistent with EGT’s core perspective focusing on the dynamic adjustment of agents’ strategies under constraints, providing practical insights for NPOs to optimize cost control strategies [12,13]. In addition, dynamic demand forecasting models [14] and human-centered deployment mechanisms have provided technical support for NPOs’ reserves planning [15]; Wood et al.used a combination of computer-supported evolutionary game theory and agent-based modeling to examine the competition between the Organization of Petroleum Exporting Countries (OPEC) and the Seven Sisters oil companies for control of the global oil market during the 1960s and 1970s [16];Yuan et al. (2023) directly applied EGT to the optimization of medical supplies allocation, further confirming the applicability and effectiveness of this theory in the research field [17]. In terms of research on emergency reserves system construction and incentive mechanism design, Xiao et al. (2022) constructed an NPO-enterprise capacity incentive model, but failed to fully incorporate the regulatory role of government policies [18]. Some studies have confirmed the potential risks associated with blind subsidies [19], while verifying the effectiveness of technology-upgrading subsidies in stimulating enterprises’ participation enthusiasm—these research conclusions can be combined with Samuelson’s (1997) heterogeneous agent framework to provide a more solid theoretical basis for explaining enterprises’ strategy selection and dynamic transformation under different incentive policies [20].

In summary, scholars have achieved fruitful outcomes in the field of emergency material reserves, with research scopes covering reserves system construction, supply chain collaborative management, and cost-benefit optimization. However, existing literature lacks in-depth exploration of the interactive game mechanism between non-profit organizations (NPOs) and reserves-holding enterprises under the framework of government incentive policies, particularly insufficient attention to the differential impacts of multiple incentive tools and the evolutionary logic of game equilibrium. Compared with existing studies, this paper conducts a theoretical modeling analysis based on evolutionary game theory. The innovations of this paper are mainly reflected in two aspects: first, it systematically examines the implementation effects of multiple collaborative policy instruments, including one-time subsidies, revenue sharing, cost sharing, and regulatory penalties, and reveals the differential impacts of different policy combinations on the strategic choices of game participants; second, it innovatively takes the cost-benefit ratio as the core analytical dimension to deconstruct the intrinsic mechanism through which changes in key parameters affect the evolutionary game equilibrium of emergency medical material production capacity reserves. Based on this, under the assumption of “bounded rationality”, this paper adopts the evolutionary game method to explore the optimal strategy choices of NPOs and reserves enterprises in emergency medical material production capacity reserves, aiming to provides new perspectives and practical guidance for optimizing the emergency medical material reserves system and ensuring the stable development of emergency reserves cooperation. The innovative aspects of this paper compared with related research are shown in Table 1.

**Table 1:** Comparison analysis between this paper and existing literature research content.

Issues Literature	This paper	[5]	[21]	[17]	[22]
NPOs and goverment incentive policies	✓			✓	
production capacity of emergency medical	✓	✓			✓
regulatory penalties	✓		✓	✓	✓

The structure of this paper is organized as follows: In Basic assumptions and model establishment section defines the game participants, clarifies the assumptions, and constructs the evolutionary game model. In Numerical simulation section analyzes the stability of the game system and the sensitivity of key parameters through numerical simulation. In Conclusions section summarizes the research conclusions and prospects future research directions.

## 2 Basic assumptions and model establishment

### 2.1 Game agents analysis

The game agents in this chapter are defined as NPOs represented by the Red Cross Society and enterprises qualified for emergency medical supplies capacity reserves. Both are randomly selected from participants of different emergency medical supplies reserves systems, and the two groups conduct multiple rounds of paired games. Based on the actual research situation, this study assumes that during the game, NPOs have two strategy choices: “entrusted reserves” or “non-entrusted reserves”, while the corresponding strategies for reserves enterprises are “reserves” or “non-reserves” [6,23]. Evolutionary Game Theory (EGT) takes bounded rationality under asymmetric information as its core assumption. As the core game participants, NPOs and reserves enterprises will seek optimal strategies under the constraint of balanced revenue and expenditure based on their own interest demands. In addition to basic benefits, their benefits structure includes two additional dimensions:first, the indirect benefits derived from reputation value and social disaster reduction value within the emergency medical supplies reserve system. Herein, reputation value [24–26]. second, additional rewards or corresponding penalties arising from differences in strategy choices under the framework of government incentive policies. Firstly, the “perfect competition” referred to in this paper denotes an operational model where, in the scenario of no direct government intervention, NPOs and reserves enterprises carry out emergency reserves-related activities through the spontaneous regulation of supply-demand matching and resource flow, with the core goal of maximizing individual interests. Their decisions and behaviors are only constrained by market signals and competitive rules. In the field of emergency medical supplies reserves, both can generate social reputation benefits by fulfilling social responsibilities and conducting emergency medical supplies capacity reserves. The scale of such reputation benefits depends on the inherent value of the reputation and the participants’ ability to convert reputation into tangible benefits. Secondly, conducting emergency medical supplies capacity reserves enables the timely provision of emergency medical materials to support rescue operations when public health emergencies occur, thereby generating social disaster reduction benefits. Such benefits are directly related to the quality of emergency medical supplies reserves, specifically including key dimensions such as the rationality of reserves varieties, the scientificity of reserves quantity and layout, and the efficiency and convenience of information transmission—these factors collectively determine the implementation effect of emergency rescue material support. Thirdly, during the process of conducting emergency medical supplies capacity reserves, both incur direct and indirect reserves costs such as production equipment reservation and inventory occupation. After offsetting such costs against the basic benefits and reputation conversion benefits from reserves activities, reserves gains and losses are ultimately formed. However, with the continuous improvement of loan interest subsidy policies [23], the fluctuation range of reserves gains and losses will be effectively suppressed, thereby reducing their operational risks. Finally, in view of the public guarantee characteristics of the emergency medical supplies reserves system, to construct this system more efficiently, the government will, in addition to relying on the perfect competition to play a leading role, formulate corresponding incentive policies (the government does not participate as an evolutionary game subject) to promote their cooperation in the field of emergency medical supplies capacity reserves and accelerate the implementation of such cooperation.

### 2.2 Basic assumption

Assumption 1: This study constructs an evolutionary game model between NPOs and enterprises with information asymmetry between the two parties as the core premise; in the game process, the strategies of both parties are dynamically adjusted through continuous interaction and gradually advanced in the cycle of trial-and-error correction [27,28].

Assumption 2: The players in the game, which include the NPOs and reserves enterprises are all finitely rational game agents who cannot always make entirely rational decisions [29].

Assumption 3: The basic benefits $\pi_{1}$ and $\pi_{2}$ that the NPOs and the reserves enterprises can respectively obtain during the cooperation process.

Assumption 4: If the NPOs choose to entrust the reserves enterprises to undertake the reserves during cooperation, they can obtain social reputation benefits [30,31] and disaster reduction benefits [26,32–33] brought by emergency reserves.The social reputation benefits is recorded as ${𝒦}_{i}R(i=1,2)$. Among them, *R* is the reputation value generated by the reserves of emergency medical supplies, and 𝒦 is the reputation return coefficient, which refers to the ability of participating entities to convert the reserves reputation of emergency medical supplies production capacity into revenue.Due to the social disaster reduction benefits brought by emergency reserves, some of the social disaster reduction benefits can be converted into profits for the NPOs and the reserves enterprises, denoted as *hB*_1_. Among them, *B*_1_ is the social disaster reduction benefits brought by the emergency medical material production capacity reserves. The higher the benefits, the greater the social welfare created by the participating entities through the emergency medical material production capacity reserves, *h* is social disaster reduction benefits coefficient. The distribution coefficient of the NPOs is 1−k, the distribution coefficient of the reserves enterprises are *k*, and k∈[0,1].

Assumption 5: When NPOs select the entrusted reserves strategy to carry out emergency medical supplies capacity reserves [34], they incur corresponding entrustment costs, denoted as *C*_1_ [23]. When medical material production enterprises choose the same capacity reserves strategy, they incur corresponding capacity reserves costs, denoted as *C*_2_. If NPOs adopt the entrusted reserves strategy while reserves enterprises adopt the rejecting reserves strategy, reserves enterprises may engage in speculative behaviors by leveraging market price fluctuations of medical materials, thereby obtaining additional benefits, denoted as *N*_2_. If NPOs adopt the non-entrusted reserves strategy while reserves enterprises adopt the reserves strategy, NPOs can purchase ready-made materials from the market to avoid entrustment costs and generate additional benefits, denoted as *N*_1_.

Assumption 6: The reserves cooperation cost is denoted as $\nu A_{i}(i=1,2)$, where *A*_1_ and *A*_2_ correspond to the opportunity costs incurred by NPOs and reserves enterprises when conducting reserves cooperation [35], respectively. ν∈[0,1] is the opportunity cost coefficient, characterizing the sensitivity of game participants to opportunity costs [36]. When ν=0, it indicates that game participants do not consider opportunity costs at all in decision-making; when ν=1, it indicates that game participants fully consider all opportunity costs.

Assumption 7: In the scenario of emergency medical supplies capacity reserves, the proportions of NPO groups choosing the entrusted and non-entrusted reserves strategies are *x* and 1−x, respectively, while the proportions of reserves enterprise groups choosing the reserves and non-reserves strategies are *y* and 1−y, respectively. Both *x* and *y* are functions of time and satisfy x,y∈[0,1].

The relevant variables and their explanations are shown in Table 2 below, involving non-negative real numbers for all relevant variables. As game theory is usually based on simplifying assumptions and theoretical models to analyze the actual problem, the dependent relationship between variables is usually not considered.

**Table 2:** Variables and explanations.

Parameter	Parameter meaning
$\pi_{0}$	The NPOs first payoff
*R*	Reputation value
$\kappa_{1}$	Reputation return coefficient of the NPOs
$\kappa_{2}$	Reputation return coefficient of reserves enterprises
*h*	Social disaster reduction benefits coefficient
*K*	Distribution coefficient of social benefits and income
*C*_1_	Commission cost
*C*_2_	Reserves enterprises capacity reserves cost
*N*_1_	Market direct procurement income of the NPOs
*N*_2_	Market speculation income of reserves enterprises
*A*_1_	Reserves cooperation cost of NPOs
*A*_2_	Reserves cooperation cost of reserves enterprises
ν	Reserves opportunity cost coefficients
*B*_1_	Social disaster reduction benefits
*B*_2_	Reserves achievement reward base
*B*_3_	Penalty base
$\chi_{1}$	Cost subsidy coefficient
$\chi_{2}$	Reward distribution coefficient for reserves enterprises
$\chi_{3}$	Punishment intensity

### 2.3 Evolutionary game under perfect competition

**Fig 1:**
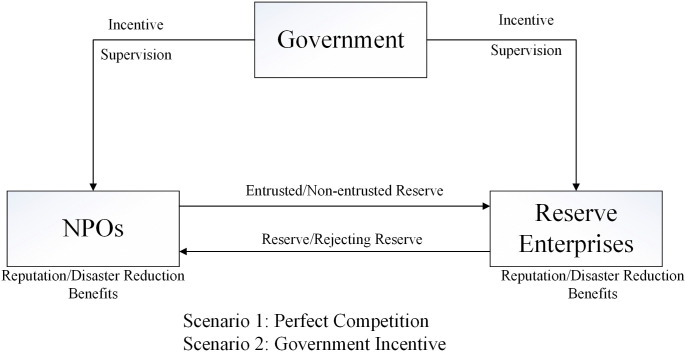
The game relationship between NPOs and reserves Enterprises.

Based on assumptions, the NPOs and the reserves enterprises engage in multiple games under perfect competition to maximize their respective interests. The game relationship between NPOs and reserves enterprises as shown in Figure 1, and the payoff matrix is shown in Table 3.

**Table 3:** Payoff matrix between NPOs and reserves enterprises.

		Reserves Enterprises	
		Reserves Strategy	Rejecting Reserves Strategy
Nonprofit organizations	Entrusted Reserves Strategy	$\pi_{1}-\pi_{0}+\kappa_{1}R+(1-k)hB_{1}-C_{1}-vA_{1}$	$\pi_{1}-C_{1}-\nu A_{1}$
		$\pi_{2}+\pi_{0}+\kappa_{2}R+khB_{1}-C_{2}-vA_{2}$	$\pi_{2}+N_{2}$
	Non-entrusted Reserves Strategy	$\pi_{1}+N_{1}$	$\pi_{1}$
		$\pi_{2}-C_{2}-\nu A_{2}$	$\pi_{2}$

The returns and average expected returns when the NPOs choose the “Entrusted Reserves Strategy” and “Non-entrusted Reserves Strategy” are $\pi_{11}$ and $\pi_{12}$, respectively.The average expected returns are $\overset{―}{\pi_{1}}$. Among them:


$$\begin{array}{cc} \pi_{11} & =y\left(\pi_{1}-\pi_{0}+\kappa_{1}R+(1-k)hB_{1}-C_{1}-vA_{1}\right)+(1-y)\left(\pi_{1}-C_{1}-vA_{1}\right)\\ & =\left[(1-k)hB_{1}+\kappa_{1}R-\pi_{0}\right]y-C_{1}-vA_{1}+\pi_{1} \end{array}$$ (1)

$$\pi_{12}=y\left(\pi_{1}+N_{1}\right)+(1-y)\pi_{1}=yN_{1}+\pi_{1}$$ (2)

$$\begin{array}{cc} {\bar{\pi }}_{1} & =x\pi_{11}+(1-x)\pi_{12}\\ & =xy\left[(1-k)hB_{1}+\kappa_{1}R-\pi_{0}-N_{1}\right]-C_{1}x-vA_{1}x+yN_{1}+\pi_{1} \end{array}$$ (3)

The replication dynamic equation of the NPOs:


$$\begin{array}{cc} F(x) & =\frac{dx}{dt}=x\left(\pi_{11}-{\bar{\pi }}_{1}\right)=x(1-x)\left(\pi_{11}-\pi_{12}\right)\\ & =x(1-x)\left\{\left[(1-k)hB_{1}+\kappa_{1}R-\pi_{0}-N_{1}\right]y-C_{1}-vA_{1}\right\} \end{array}$$ (4)

The returns and average expected returns when reserves enterprises choose the “reserves Strategy” and “Rejecting reserves Strategy” are $\pi_{21}$ and $\pi_{22}$, respectively. The average expected returns are $\bar{\pi_{2}}$. Among them:


$$\pi_{21}=x\left(\pi_{2}+\pi_{0}+\kappa_{2}R+khB_{1}-C_{2}-vA_{2}\right)+(1-x)\left(\pi_{2}-C_{2}-vA_{2}\right)$$ (5)

$$\pi_{22}=x\left(\pi_{2}+N_{2}\right)+(1-x)\pi_{2}=\pi_{2}+xN_{2}$$ (6)

$$\begin{array}{cc} {\bar{\pi }}_{2} & =y\pi_{21}+(1-y)\pi_{22}\\ & =\left(\pi_{0}+\kappa_{2}R+khB_{1}-N_{2}\right)xy+N_{2}x+\pi_{2}-C_{2}y-vA_{2}y \end{array}$$ (7)

The replication dynamic equation of the reserves enterprises:


$$\begin{array}{cc} F(y) & =\frac{dy}{dt}=y\left(\pi_{21}-{\bar{\pi }}_{2}\right)=y(1-y)\left(\pi_{21}-\pi_{22}\right)\\ & =y(1-y)\left\{\left(\pi_{0}+\kappa_{2}R+khB_{1}-N_{2}\right)x-C_{2}-vA_{2}\right\} \end{array}$$ (8)

Let *F*(*x*) = 0,*F*(*y*) = 0. There are five partial equilibrium points between the NPOs and the reserves enterprises in $D^{*}=\{(x,y)|0\leq x\leq 1,0\leq y\leq 1\}$, namely $O(0,0),A(1,0),B(1,1),C(0,1),D(x^{*},y^{*})$. Where $x^{*}=\frac{UA_{2}+C_{2}}{\pi_{0}+\kappa_{2}R+khB_{1}-N_{2}}$, $y^{*}=-\frac{\omega A_{1}+C_{1}}{khB_{1}-hB_{1}-\kappa_{1}R+\pi_{0}+N_{1}}$.

By calculating the partial derivatives of *F*(*x*) and *F*(*y*) with respect to *x* and *y* respectively, the *Jacobian* matrix of the emergency medical supplies production capacity reserves game system can be obtained as shown in (9):


$$\begin{array}{c} J=[\begin{array}{cc} \left(1-2x)\{[1-k)hB_{1}+\kappa_{1}R-\pi_{0}-N_{1}]y-C_{1}-uA_{1}\right\} & x(1-x)[(1-k)hB_{1}+\kappa_{1}R-\pi_{0}-N_{1}]\\ y(1-y)(\pi_{0}+\kappa_{2}R+khB_{1}-N_{2}) & \left(1-2y)\{(\pi_{0}+\kappa_{2}R+khB_{1}-N_{2})x-C_{2}-uA_{2}\right\} \end{array}] \end{array}$$ (9)

When *Jacobian* matrix satisfies *Det*(*J*) > 0 and *Tr*(*J*) < 0, the local equilibrium point is the stable strategy of the system [37]. This means that $0\leq \upsilon A_{2}+C_{2}\leq \pi_{0}+\kappa_{2}R+khB_{1}-N_{2}$ and $0\leq \upsilon A_{1}+C_{1}\leq (1-k)hB_{1}+\kappa_{1}R-\pi_{0}-N_{1}$, there are five local equilibrium points in the capacity reserves game system, as shown in Table 4.

**Table 4:** Stability analysis of local equilibrium points under perfect competition.

equilibrium	*Det*(*J*)	*Tr*(*J*)	Local stability
*O*(0,0)	+	–	ESS
*A*(0,1)	+	+	Unstable
*B*(1,1)	+	–	ESS
*C*(1,0)	+	+	Unstable
$D^{*}=(x^{*},y^{*})$	–	0	Saddle point

**Fig 2:**
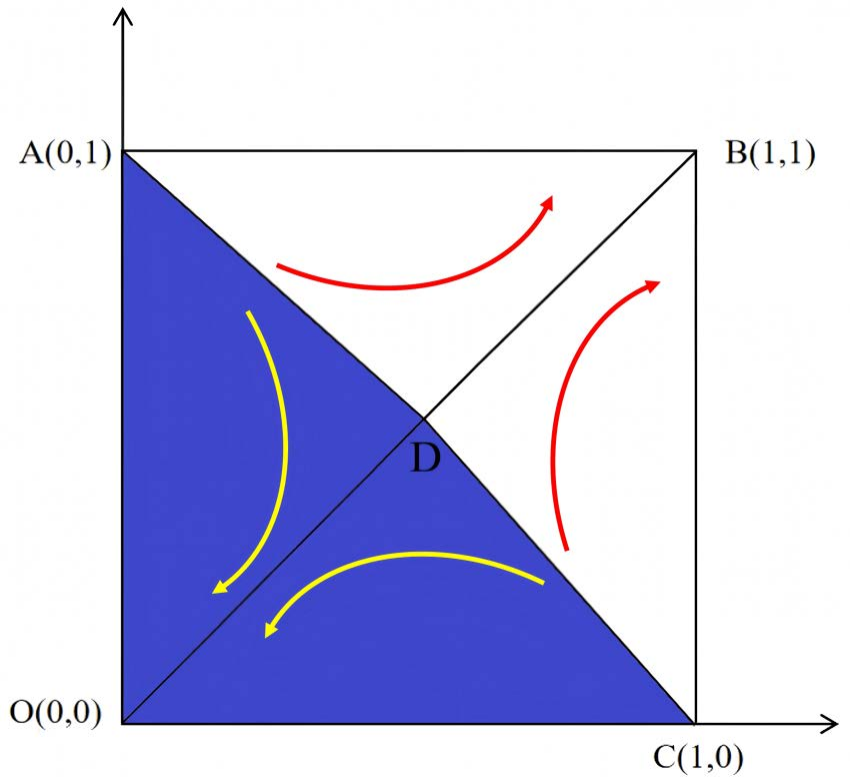
The area of S1 before evolution.

According to Figure 2, regardless of how the NPOs and the reserves enterprises make initial decisions, after multiple rounds of repeated games, the final strategy will evolve towards either direction *B*(1,1) or *O*(0,0). That is, the NPOs choose to entrust while the reserves enterprises choose to reserves, or the NPOs choose non-entrust reserves while the reserves enterprises choose rejecting reserves. The relative area size of *S*_*AOCD*_ and *S*_*ABCD*_ can reflect the final strategic choice of the NPOs and the reserves enterprises. By analyzing the factors that cause changes in area, we can infer the impact of each factor on the evolution of the game system. Let *S*_*AOCD*_ is *S*_1_, the expression is:


$$S_{1}=\frac{1}{2}(x^{*}+y^{*})=\frac{1}{2}(\frac{\upsilon A_{2}+C_{2}}{\pi_{0}+\kappa_{2}R+khB_{1}-N_{2}}-\frac{\upsilon A_{1}+C_{1}}{khB_{1}-hB_{1}-\kappa_{1}R+\pi_{0}+N_{1}})$$ (10)

**Fig 3:**
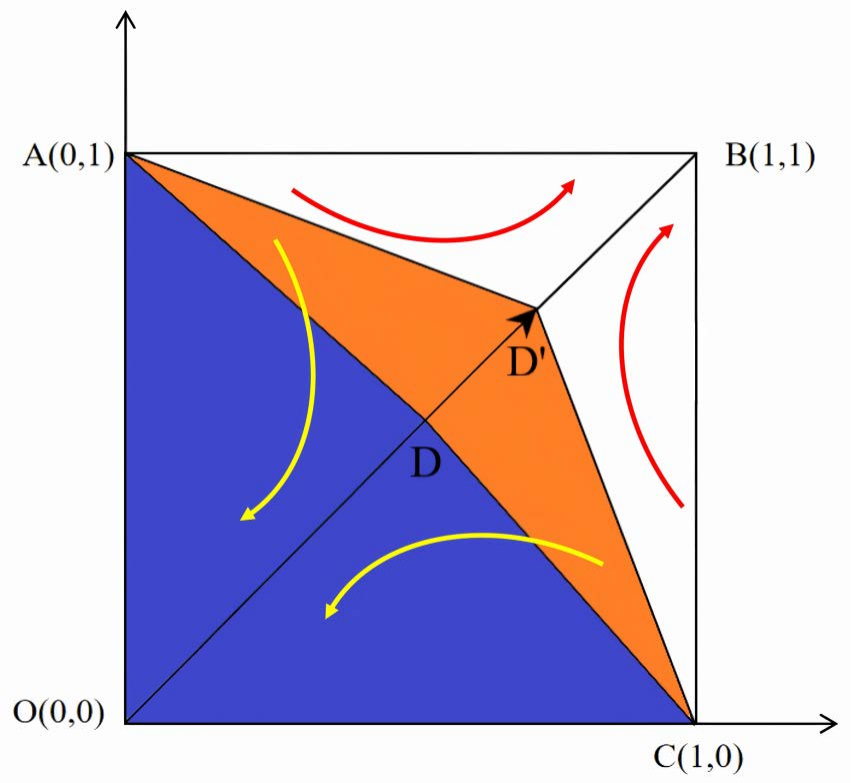
Evolutionary phase diagram of D moving towards point B.

**Fig 4:**
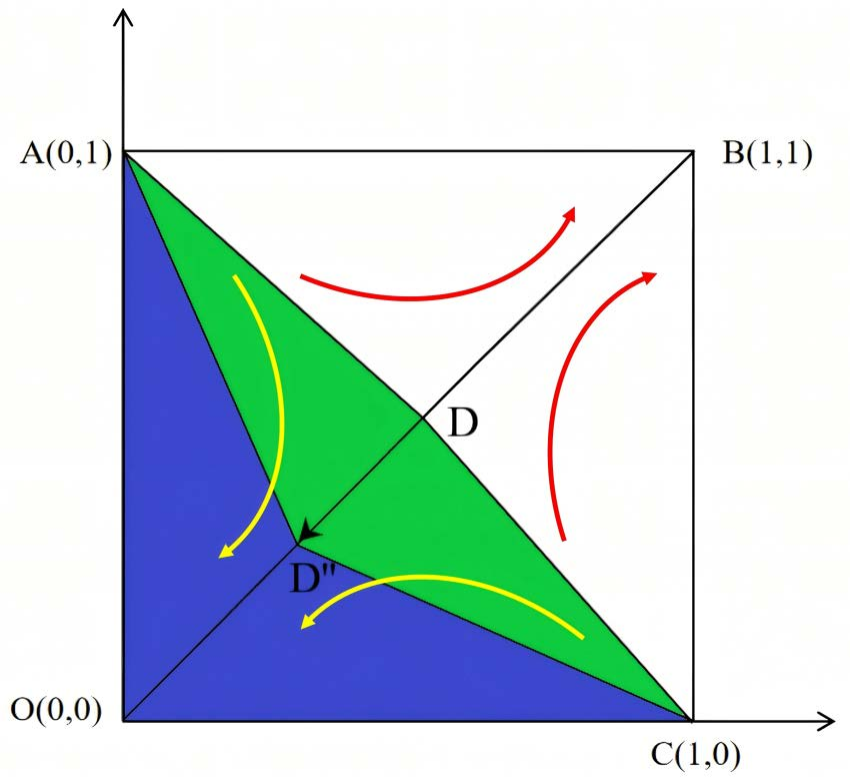
Evolutionary phase diagram of D moving towards point O.

It can be seen that under the perfect competition, *A*(0,1) and *C*(1,0) are unstable fixed points, while *O*(0,0) and *B*(1,1) are the evolutionary stable strategies of the NPOs and the reserves enterprises. Figure 2 shows the phase diagram of the evolutionary game between the NPOs and the reserves enterprises under the perfect competition. Figure 3 shows that as certain parameters increase, D shifts toward B, becoming *D*’. This indicates an increase in the area of S1 and an increased probability of the system evolving toward the O strategy (Non-entrusted, Rejecting reserves). Figure 4 shows that as certain parameters increase, D shifts toward O, becoming *D*″. This indicates an decrease in the area of S1 and an increased probability of the system evolving toward the B strategy (Entrusted, reserves).

**Proposition 1.** *Under perfect competition, the probability of the NPOs choosing to entrust and the reserves enterprises choosing to reserves decreases with increasing costs.*

*Proof.* According to $\frac{\partial S_{1}}{\partial C_{1}}=\frac{1}{2}[\frac{1}{(1-k)hB_{1}+\kappa_{1}R-N_{1}-\pi_{0}}]$, since $x^{*}$ and $y^{*}$ both belong to $D^{*}=\{(x,y)|0\leq x\leq 1,0\leq y\leq 1\}$, $\frac{\partial S_{1}}{\partial C_{1}}=\frac{1}{2}[\frac{1}{(1-k)hB_{1}+\kappa_{1}R-N_{1}-\pi_{0}}]>0$. $\frac{\partial S_{1}}{\partial C_{2}}=\frac{1}{2khB_{1}+2\kappa_{2}R-2N_{2}+2\pi_{0}}>0$ are the same, so *S*_1_ increases with the increase of *C*_1_ and *C*_2_. Therefore, the investment costs of both the NPOs and the reserves enterprises are in a single increasing relationship with the area of *S*_1_. It can be seen that *S*_*ABCD*_ will decrease with the increase of costs, and the probability of the system evolving towards *O*(0,0). the NPOs and the reserves enterprises will choose not to entrust or reserves. Proposition 1 confirmed.

Proposition 1 indicates that the production capacity reserves of emergency medical supplies is a component of the emergency system. The cost recovery cycle of its capacity reserves is relatively long. When the reserves cost is high, it will lead to a decrease in the willingness of both parties to carry out the production capacity reserves of emergency medical supplies. In practice, the high reserves cost not only increases the economic burden but also may cause enterprises to be cautious about participating in the reserves due to the inability to see obvious economic returns in the short term. Similarly, when facing limited financial resources and diverse public service demands, the government also needs to make a trade-off between emergency material reserves and other public expenditures.

**Proposition 2.** *Under the dominance of the perfect competition, the probability that reserves enterprises choose the reserves strategy increases with the reduction of reserves cooperation costs and reserves opportunity cost coefficients.*

*Proof.* From $\frac{\partial S_{1}}{\partial A_{1}}=\frac{1}{2}[\frac{1}{(1-k)hB_{1}+\kappa_{1}R-N_{1}-\pi_{0}}]>0$, it can be inferred that $\frac{\partial S_{1}}{\partial A_{2}}>0$ has $\frac{\partial S_{1}}{\partial \upsilon }=\frac{1}{2}\{\frac{[(1-k)hB_{1}+\kappa_{1}R-N_{1}-\pi_{0}]A_{2}+(khB_{1}+\kappa_{2}R-N_{2}+\pi_{0})A_{1}}{\left[(1-k)hB_{1}+\kappa_{1}R-N_{1}-\pi_{0}](khB_{1}+\kappa_{2}R-N_{2}+\pi_{0}\right)}\}>0$.Therefore, *S*_1_ increases with the increase of ν, *A*_1_ and *A*_2_, while the area of *S*_1_ decreases with the decrease of ν, *A*_1_ and *A*_2_. At this point, the system evolves towards *B*(1,1). Proposition 2 confirmed.

Proposition 2 indicates that the investment scale of emergency medical supplies capacity reserves has a significant impact on both entrusting parties and reserves enterprises. The dual existence of reserves cooperation costs and reserves opportunity cost coefficients may reduce the probability that NPOs and reserves enterprises carry out emergency reserves. This is because, facing high capacity investment, potential loss risks, and opportunity cost pressures, both parties will be more cautious in deciding to participate in emergency reserves, and their concerns mainly stem from the uncertainty of cost recovery and considerations of opportunity costs. However, third-party financial policies such as interest subsidies can specifically alleviate the constraints of reserves opportunity cost coefficients and effectively reduce enterprises’ concerns: by reducing financing costs, they not only ease direct economic pressure but also indirectly offset the implicit pressure brought by opportunity costs, thereby enhancing enterprises’ enthusiasm for participating in emergency medical capacity reserves. Specifically, interest subsidies can directly reduce financing costs, reduce the opportunity cost loss caused by fund occupation, and further alleviate the burden on participants. Therefore, when promoting emergency medical supplies capacity reserves work, NPOs should fully consider the dual impacts of the two types of costs, implement third-party financial policies such as interest subsidies to reduce cost-related concerns and improve participants’ willingness and enthusiasm for reserves; meanwhile, strengthen policy publicity to promote more participants to understand and use policy tools, and jointly promote the smooth development of emergency medical supplies capacity reserves work. Ultimately, the system will steadily evolve toward the evolutionary path of “Entrusted reserves – reserves.”

**Proposition 3.** *Under the scenario dominated by the perfect competition, the higher the social disaster reduction benefits coefficient, the more significant the social disaster reduction benefits generated by the reserves of emergency medical material production capacity, and the corresponding social welfare level will also be higher. Consequently, the benefits obtainable by the participating agents (NPOs and reserves enterprises) will increase accordingly. Therefore, the probability that NPOs choose to entrust reserves will increase, and the probability that reserves enterprises opt to carry out reserves will also rise synchronously.*

*Proof.* From $\frac{\partial S_{1}}{\partial h}=-\frac{2(\omega A_{2}+C_{2})kB_{1}}{{\left(2khB_{1}+2\kappa_{2}R-2N_{2}+2\pi_{0}\right)}^{2}}-\frac{\left(\omega A_{1}+C_{1})(2B_{1}-2kB_{1}\right)}{{\left[(1-k)2hB_{1}+2\kappa_{1}R-2N_{1}-2\pi_{0}\right]}^{2}}<0$, it can be inferred that $\frac{\partial S_{1}}{\partial B_{1}}<0$ also indicates that *S*_1_ decreases with the increase of *h* and *B*_1_, indicating that the system is evolving towards *B*(1,1). Proposition 3 confirmed. □

Proposition 3 indicates that with the improvement of social disaster reduction benefits coefficient and the increase of social disaster reduction benefits, both parties are more inclined to choose entrusted and reserves strategies. Especially when the production capacity of emergency medical supplies is reasonably optimized in terms of material categories and layout, the social disaster reduction benefits generated will be significantly improved. This scientific reserves method not only provides timely critical material support for rescue operations, but also brings higher profits to NPOs and reserves enterprises, and enhances their social reputation and brand image. More importantly, this optimization strategy enhances the willingness of both parties to cooperate, helps to form a stable cooperative relationship, and thus increases the support of production capacity reserves for emergency rescue. This positive interaction and cooperation further promote the overall development of emergency medical material production capacity reserves, providing important guarantees for social stability and sustainable development.

**Proposition 4.** *Under perfect competition, as the conversion coefficient of reputation benefits and reputation value increase, the probability of the receiving enterprises choosing to reserves increases.*

*Proof.* From $\frac{\partial S_{1}}{\partial \kappa_{1}}=-\frac{2(\upsilon A_{1}+C_{1})R}{{\left[(1-k)2hB_{1}+2\kappa_{1}R-2N_{1}-2\pi_{0}\right]}^{2}}<0$, it can be inferred that $\frac{\partial S_{1}}{\partial \kappa_{2}}<0$ and $\frac{\partial S_{1}}{\partial R}<0$. Therefore, *S*_1_ decreases as $\kappa_{1}$, $\kappa_{2}$, and *R* increase, indicating that the system is evolving towards *B*(1,1). Proposition 4 confirmed.□

Proposition 4 indicates that by carrying out emergency medical material production capacity reserves, both the NPOs and the reserves enterprises can not only fulfill their social responsibilities and provide strong support for responding to emergency situations, but also gain reputation value as a result. In this process, both parties can enhance their ability to convert reputation benefits by fully leveraging their respective characteristics and advantages, such as the commissioning party’s organizational and coordination capabilities and the reserves party’s professional production and reserves management level. The enhancement of this conversion ability further increases the reputation benefits of both parties, such as gaining widespread recognition and respect from society, thereby enhancing brand image and market position. Therefore, when they see such reputation value and potential benefits, NPOs and reserves companies will be more willing to choose entrusted and reserves strategies, as this not only benefits society but also brings long-term benefits to themselves.

**Proposition 5.** *Under perfect competition, the probability of deposit taking enterprises choosing not to deposit increases with the increase of speculative returns.*

*Proof.* From $\frac{\partial S_{1}}{\partial N_{1}}=\frac{2(\omega A_{1}+C_{1})}{{\left[(1-k)2hB_{1}+2\kappa_{1}R-2N_{1}-2\pi_{0}\right]}^{2}}>0$, the same goes for $\frac{\partial S_{1}}{\partial N_{2}}>0$. Therefore, *S*_1_ increases with the increase of *N*_1_ and *N*_2_, and the system evolves towards *O*(0,0), which is confirmed.□

Proposition 5 indicates that the production capacity reserves enterprises that choose to reserves will have to bear the risks brought by price fluctuations. When emergency events occur, they will still sell according to the agreed price of the entrusted agent. If they do not participate in the reserves reserves enterprises, they will obtain speculative returns based on market premiums; If the reserves enterprises independently carries out reserves based on market experience, and the NPOs does not entrust, then the NPOs can purchase from the market in case of emergency events, thereby avoiding the cost of emergency reserves. So the reserves enterprises hopes that the NPOs choose the entrustment strategy and enjoys the market premium income. The NPOs hopes that the reserves enterprises choose to reserves while avoiding the cost of emergency medical material production capacity reserves. When the two speculative profits increase, it will cause dissatisfaction among the participants in the reserves of emergency medical supplies production capacity. This dissatisfaction will lead the NPOs and the reserves enterprises to evolve towards a combination of non commissioning and non reserves strategies, respectively.

**Proposition 6.** *Under perfect competition, the final decision of both parties in the game is influenced by the distribution coefficient of social benefits from reserves, which depends on the specific situation.*

*Proof.* From $\frac{\partial S_{1}}{\partial k}=-\frac{2(\omega A_{2}+C_{2})hB_{1}}{{\left(2khB_{1}+2\kappa_{2}R-2N_{2}+2\pi_{0}\right)}^{2}}+\frac{2(\omega A_{1}+C_{1})hB_{1}}{{\left[(1-k)2hB_{1}+2\kappa_{1}R-2N_{1}-2\pi_{0}\right]}^{2}}$, it can be inferred that the relationship between *k* and *S*_1_ depends on the situation. When $\frac{[(1-k)2hB_{1}+2\kappa_{1}R-2N_{1}-2\pi_{0}{]}^{2}}{(2khB_{1}+2\kappa_{2}R-2N_{2}+2\pi_{0}{)}^{2}}>\frac{(\upsilon A_{1}+C_{1})hB_{1}}{(\upsilon A_{2}+C_{2})hB_{1}}$ there is $\frac{\partial S_{1}}{\partial k}<0$, and *S*_1_ decreases with the increase of *k*. Therefore, it can be inferred that in the current situation, the system evolves towards *B*(1,1) with the increase of the profits distribution coefficient; When $\frac{[(1-k)2hB_{1}+2\kappa_{1}R-2N_{1}-2\pi_{0}{]}^{2}}{(2khB_{1}+2\kappa_{2}R-2N_{2}+2\pi_{0}{)}^{2}}<\frac{(\upsilon A_{1}+C_{1})hB_{1}}{(\upsilon A_{2}+C_{2})hB_{1}}$, there is $\frac{\partial S_{1}}{\partial k}>0$, and *S*_1_ increases with the increase of *k*. It can be inferred that under this condition, an increase in the profits distribution coefficient *k* will cause the system to evolve towards *O*(0,0). □

Proposition 6 indicates that under perfect competition, the final decision of both parties in the game is influenced by the distribution coefficient of social benefits from reserves, which depends on the specific situation. In practice, the final decision of both parties in the game is indeed influenced by the distribution coefficient of social benefits from reserves. This impact is not fixed and unchanging. Specifically, the setting and adjustment of distribution coefficients can directly affect the profits distribution pattern of the NPOs and the reserves enterprises in the reserves of emergency medical supplies production capacity. When the allocation coefficient is set more reasonably and fairly, it can motivate both parties to actively participate in reserves work and jointly promote the implementation of reserves strategies. This is because a reasonable allocation coefficient can ensure that both parties receive corresponding returns in cooperation, reduce cooperation risks, and enhance cooperation confidence. However, if the allocation coefficient is set improperly or there is controversy, it may lead to conflicts and differences of interest between the NPOs and the reserves enterprises, thereby affecting their willingness and decision-making to participate in the reserves strategy. Therefore, under the perfect competition, it is necessary to fully consider and reasonably set the distribution coefficient of reserves social benefits to promote cooperation and win-win outcomes between both parties in the game.

### 2.4 Evolutionary Game under Government Incentive Policies

Governments attach great importance to the reserves of emergency medical supplies and actively promote the construction of the emergency reserves system. In this process, governments, on the one hand, provide diversified policy incentives for the development of the emergency reserves system, and on the other hand, continuously strengthen the intensity of supervisory and disciplinary measures. Through the dual mechanism of “incentives + constraints”, they create a favorable and fair development environment for the orderly construction of the emergency reserves system.

Assumption 8: To advance the development of emergency reserves systems, reserves enterprises should take on greater social responsibility—thus encouraging their participation in the reserves of production capacity for emergency medical supplies [38]. The achievement reward for the reserves enterprises are $\chi_{2}B_{2}$. Where *B*_2_ rewards the reserves achievements that meet the conditions, $\chi_{2}\in [0,1]$ is the distribution coefficient of the reserves NPOs. When the NPOs does not entrust the enterprises to reserves, but the enterprises actively carries out corresponding emergency medical material reserves, the NPOs can provide a reserves cost subsidy to the enterprises, which is recorded as $\chi_{1}C_{2}$. Where $\chi_{1}\in [0,1]$ represents the cost subsidy intensity of the NPOs. In addition, governments conduct investigations on enterprises with potential reserves strength, and screen out potential high-quality reserves enterprises. For enterprises listed in the potential reserves list of emergency medical material production capacity, they entrust reserves services. If the listed enterprises choose not to reserves, a fine $\chi_{3}B_{3}$ will be imposed, where $\chi_{3}$ is the severity of the punishment. The profits matrix of both parties is shown in Table 5. The solving process is similar to that under perfect competition. According to the payoff matrix of the NPOs and the reserves enterprises under the incentive policy in Table 5, the replicative dynamic equations of the NPOs and the reserves enterprises can be obtained.

**Table 5:** Payoff matrix of NPOs and reserves enterprise under incentive policies.

		Reserves Enterprises	
		Reserves Strategy	Rejecting Reserves Strategy
Nonprofit organizations	Entrusted Reserves Strategy	$\pi_{1}-\pi_{0}+\kappa_{1}R+(1-k)hB_{1}-C_{1}-\upsilon A_{1}+(1-\chi_{2})B_{2}$	$\pi_{1}-C_{1}-\upsilon A_{1}$
		$\pi_{2}+\pi_{0}+\kappa_{2}R+khB_{1}-C_{2}-uA_{2}+\chi_{2}B_{2}$	$\pi_{2}+N_{2}-\chi_{3}B_{3}$
	Non-entrusted Reserves Strategy	$\pi_{1}+N_{1}-\chi_{1}C_{2}$	$\pi_{1}$
		$\pi_{2}-C_{2}-\nu A_{2}+\chi_{1}C_{2}$	$\pi_{2}$

The return $\pi_{11}^{\prime }$ when the NPOs choose the “Entrusted reserves Strategy”, the return $\pi_{12}^{\prime }$ when choosing the “Non-entrusted reserves Strategy”, and the average expected return $\pi_{1}^{\prime }$ are respectively:


$$\begin{array}{cc} \pi_{11}^{\prime }= & y[\pi_{1}-\pi_{0}+\kappa_{1}R+(1-k)hB_{1}-C_{1}-uA_{1}+(1-\chi_{2})B_{2}]\\ & +(1-y)(\pi_{1}-C_{1}-uA_{1})\\ = & [(1-k)hB_{1}+\kappa_{1}R-\pi_{0}+(1-\chi_{2})B_{2}]y-C_{1}-uA_{1}+\pi_{1} \end{array}$$ (11)

$$\pi_{12}^{\prime }=y(\pi_{1}+N_{1}-\chi_{1}C_{2})+(1-y)\pi_{1}=y(N_{1}-\chi_{1}C_{2})+\pi_{1}$$ (12)

$$\begin{array}{cc} {\overset{―}{\pi^{\prime }}}_{1}= & x{\pi^{\prime }}_{11}+(1-x){\pi^{\prime }}_{12}\\ = & xy[\chi_{1}C_{2}+(1-k)hB_{1}+(1-\chi_{2})B_{2}+\kappa_{1}R-\pi_{0}-N_{1}]\\ & -C_{1}x-\upsilon A_{1}x+(N_{1}-\chi_{1}C_{2})y+\pi_{1} \end{array}$$ (13)

The replicator dynamics equation for the NPOs is:


$$F^{'}(x)=\frac{dx}{dt}=x(\pi^{'}{\;}_{11}-\bar{\pi^{'}}{\;}_{1})\;=x(1-x)\{[\chi_{1}C_{2}+(1-k)hB_{1}+(1-\chi_{2})B_{2}+\kappa_{1}R-\pi_{0}-N_{1}]y-C_{1}-\upsilon A_{1}\}$$ (14)

Similarly, the benefit $\pi_{21}^{\prime }$ for the reserves enterprises when choosing the “reserves Strategy” is:

The benefits of choosing the ’Rejecting reserves Strategy’ are:


$$\pi_{22}^{\prime }=\pi_{2}+x(N_{2}-\chi_{3}B_{3})$$ (15)

The average expected return ${\overset{―}{\pi^{\prime }}}_{2}$ is:


$$\begin{array}{cc} {\overset{―}{\pi^{\prime }}}_{2}= & (khB_{1}+\chi_{2}B_{2}+\chi_{3}B_{3}-\chi_{1}C_{2}+\kappa_{2}R-N_{2}+\pi_{0})xy\\ & -(1-\chi_{1})C_{2}y-\upsilon A_{2}y+(N_{2}-\chi_{3}B_{3})x+\pi_{2} \end{array}$$ (16)

Then there is, the replication dynamic equation of the reserves enterprises is:


$$F^{'}(y)=\frac{dy}{dt}=y(\pi^{'}{\;}_{21}-\bar{\pi^{'}}{\;}_{2})\;=y(1-y)[(khB_{1}+\chi_{2}B_{2}+\chi_{3}B_{3}-\chi_{1}C_{2}+\kappa_{2}R-N_{2}+\pi_{0})x-(1-\chi_{1})C_{2}-uA_{2}]$$ (17)

*O*(0,0),*A*(1,0),*B*(1,1),*C*(0,1) and $D^{\prime }(x^{*},y^{*})$ are the five local equilibrium points of $F^{\prime }(x)=0$ and $F^{\prime }(y)=0$ within region $D^{\prime^{*}}=\{(x,y)|0\leq x\leq 1,0\leq y\leq 1\}$. The horizontal and vertical coordinates of point $D^{\prime }(x^{*},y^{*})$ is $x^{*}=\frac{\upsilon A_{2}-\chi_{1}C_{2}+C_{2}}{khB_{1}+\chi_{2}B_{2}+\chi_{3}B_{3}-\chi_{1}C_{2}+\kappa_{2}R-N_{2}+\pi_{0}}$, $y^{*}=-\frac{\omega A_{1}+C_{1}}{khB_{1}-hB_{1}+\chi_{2}B_{2}-\chi_{1}C_{2}+\kappa_{1}R-B_{2}+N_{1}+\pi_{0}}$. Further obtaining the partial derivatives of $F^{\prime }(x)$ and $F^{\prime }(y)$ with respect to *x*1 and *y*, the Jacobian matrix $J^{\prime }$ of the game system can be obtained.


$$\begin{array}{c} J=[\begin{array}{cc} (1-2x)\{[\chi_{1}C_{2}+(1-k)hB_{1}+(1-\chi_{1})B_{2} & x(1-x)[\chi_{1}C_{2}+(1-k)hB_{1}\\ +\kappa_{1}R+-\pi_{0}-N_{1}]y-C_{1}-uA_{1}\} & +(1-\chi_{1})B_{2}+\kappa_{1}R-\pi_{0}-N_{1}]\\ y(1-y)(khB_{1}+\chi_{2}B_{2}+\chi_{3}B_{3} & (1-2y)[(khB_{1}+\chi_{2}B_{2}+\chi_{3}B_{3}-\chi_{1}C_{2}+\kappa_{2}R\\ -\chi_{1}C_{2}+\kappa_{2}R+\pi_{0}-N_{2}) & +\pi_{0}-N_{2})x-(1-\chi_{1})C_{2}-uA_{2} \end{array}] \end{array}$$ (18)

When the values and traces of the Jacobian matrix satisfy $Det(J^{\prime })>0$ and $Tr(J^{\prime })<0$, the evolutionary stable strategy ESS can be obtained [37]. When $0<x^{*},y^{*}<1$, the game system under this incentive policy has 5 local equilibrium points, as shown in Table 6.

**Table 6:** Stability analysis of local equilibrium points under incentive policies.

equilibrium	*Det*(*J*)	*Tr*(*J*)	Local stability
*O*(0,0)	+	–	ESS
*A*(0,1)	+	+	Unstable
*B*(1,1)	+	–	ESS
*C*(1,0)	+	+	Unstable
$D^{\prime }(x^{*},y^{*})$	–	0	Saddle point

According to Table 6, after introducing incentive policy regulation, the saddle point, unstable point, and stable point of the game system can be obtained. The above local stable points are consistent with the perfect competition background. *O*(0,0) and *B*(1,1) are evolutionary equilibrium stable strategies, indicating that the evolutionary phase diagram under incentive policy and the phase diagram under perfect competition have not changed.

Further research on the relationship between various influencing factors can be conducted by analyzing the relative sizes of *S*_*AOCD*_ and *S*_*ABCD*_. *S*_*AOCD*_ is denoted as *S*_2_, then:


$$\begin{array}{cc} S_{2}= & \frac{1}{2}(x^{*}+y^{*})\\ = & \frac{1}{2}(\frac{\upsilon A_{2}+\chi_{1}C_{2}+C_{2}}{khB_{1}+\chi_{2}B_{2}+\chi_{3}B_{3}-\chi_{1}C_{2}+\kappa_{2}R-N_{2}+\pi_{0}}\\ & -\frac{\upsilon A_{1}+C_{1}}{khB_{1}-hB_{1}+\chi_{2}B_{2}-\chi_{1}C_{2}+\kappa_{1}R-B_{2}+N_{1}+\pi_{0}}) \end{array}$$ (19)

**Proposition 7.** *The probability of a reserves enterprises choosing a reserves strategy increases with the increase in cost subsidies from the NPOs and rewards for production capacity reserves achievements.*

*Proof.* From $0<x^{\prime^{*}}<1$ and $\frac{\partial S_{2}}{\partial \chi_{1}}=-\frac{2(khB_{1}+\chi_{2}B_{2}+\chi_{3}B_{3}+\kappa_{2}R+\pi_{0}-N_{2}-\upsilon A_{2}-C_{2})C_{2}}{(2khB_{1}+2\chi_{2}B_{2}+2\chi_{3}B_{3}-2\chi_{1}C_{2}+2\kappa_{2}R-2N_{2}+2\pi_{0}{)}^{2}}-\frac{2(\upsilon A_{1}+C_{1})C_{2}}{(2khB_{1}-2hB_{1}+2\chi_{2}B_{2}-2\chi_{1}C_{2}-2\kappa_{1}R-2B_{2}+2N_{1}+2\pi_{0}{)}^{2}}$, there is $\frac{\partial S_{2}}{\partial \chi_{1}}<0$. Similarly, according to $\frac{\partial S_{2}}{\partial B_{2}}=-\frac{2(\upsilon A_{2}-\chi_{1}C_{2}+C_{2})\chi_{2}}{(2khB_{1}+2\chi_{2}B_{2}+2\chi_{3}B_{3}-2\chi_{1}C_{2}+2\kappa_{2}R-2N_{2}+2\pi_{0}{)}^{2}}-\frac{2(\upsilon A_{1}+C_{1})(1-\chi_{2})}{(2khB_{1}-2hB_{1}+2\chi_{2}B_{2}-2\chi_{1}C_{2}-2\kappa_{1}R-2B_{2}+2N_{1}+2\pi_{0}{)}^{2}}$, there is $\frac{\partial S_{2}}{\partial B_{2}}<0$. So as $\chi_{1}$ and *B*_2_ increase, the area of *S*_2_ decreases, and the game system under incentive policies evolves towards *B*(1,1).Proposition 7 is proven.*□*

Proposition 7 indicates that the reserves of emergency medical supplies production capacity, as an important component of the goverment emergency system construction, is the vanguard of social responsibility. The benefits of its reserves are directly related to the social benefits of emergency rescue. Therefore, cost subsidies and reserves achievement rewards have become important incentive measures. These two measures can effectively reduce the costs of reserves enterprises and increase their profits, thereby playing a positive role in promoting the selection of emergency medical material production capacity reserves strategies by enterprises. Through this positive incentive, more enterprises can be encouraged to participate in the reserves of emergency medical supplies production capacity, contributing to the smooth implementation of goverment emergency rescue work.

**Proposition 8.** *The probability of reserves enterprises choosing to reserves increases with the increase of the penalty base and severity.*

*Proof.* From (19), $\frac{\partial S_{2}}{\partial \chi_{3}}=-\frac{2(\upsilon A_{2}-\chi_{1}C_{2}+C_{2})B_{3}}{(2khB_{1}+2\chi_{2}B_{2}+2\chi_{3}B_{3}-2\chi_{1}C_{2}+2\kappa_{2}R-2N_{2}+2\pi_{0}{)}^{2}}$, then $\frac{\partial S_{2}}{\partial \chi_{3}}>0$. Similarly $\frac{\partial S_{2}}{\partial B_{3}}>0$. So the area of *S*_2_ increases with the increase of $\chi_{3}$ and *B*_3_, and the system evolves towards *O*(0,0). Proposition 8 is confirmed.□

Proposition 8 indicates that setting a reasonable penalty base and severity can significantly increase the cost of non-compliance for reserves enterprises in emergency medical supplies reserves, effectively reducing their motivation for speculative behavior. With the gradual strengthening of punishment, reserves enterprises will pay more attention to avoiding risks such as economic losses, reputation damage, and potential legal liabilities. Therefore, they are more inclined to choose a prudent reserves strategy rather than taking risks in speculative behavior. This institutional design not only helps regulate the behavior of reserves enterprises, but also enhances the stability and reliability of the entire emergency medical material reserves system, providing a solid guarantee for medical material production capacity reserves in response to emergencies.

**Proposition 9.** *The impact of the size of* $\chi_{2}$ *on the outcome of the game depends on the specific situation.*

*Proof.* When $(\frac{khB_{1}+\chi_{2}B_{2}+\chi_{3}B_{3}-\chi_{1}C_{2}+\kappa_{2}R-N_{2}+\pi_{0}}{khB_{1}-hB_{1}+\chi_{2}B_{2}-\chi_{1}C_{2}-\kappa_{1}R-B_{2}+N_{1}+\pi_{0}}{)}^{2}<\frac{\upsilon A_{2}-\chi_{1}C_{2}+C_{2}}{\upsilon A_{1}+C_{1}}$, it can be concluded that the area of $\frac{\partial S_{2}}{\partial \chi_{2}}<0$ and *S*_2_ decreases with the increase of $\chi_{2}$. When the profits sharing ratio of the reserves enterprises increase, the system will evolve towards a combination of entrusted reserves strategies. Proposition 9 is confirmed.□

Proposition 9 indicates that as the proportion of achievement rewards and benefits shared by reserves enterprises increase, the probability of the system evolving towards a combination of non commissioned and non reserved strategies may increase. This phenomenon reveals that under goverment incentive policies, the final decision of both parties in the game is indeed influenced by the allocation coefficient of the reserves enterprises, and this influence is not fixed, but varies according to specific circumstances. Specifically, the increase in the proportion of achievement rewards and profits sharing means that the reserves enterprises can obtain more economic returns when participating in the reserves of emergency medical supplies production capacity. This should have been an incentive factor to encourage the reserves enterprises to participate more actively in reserves work. However, if this increase in proportion does not fully reflect the actual contribution and risk of the reserves enterprises, or is not coordinated with other incentive measures, it may lead to a decrease in the willingness of the reserves enterprises to participate in the reserves. In addition, when faced with an increase in the proportion of rewards and benefits shared by the reserves enterprises, the NPOs may also reassess their own benefits and risks. If the NPOs believe that increasing the profits sharing ratio of the reserves enterprises will result in damage to their own interests, or that the social benefits brought by the reserves work have not met expectations, they may tend to choose a strategy of not entrusting. Therefore, when formulating goverment incentive policies, it is necessary to comprehensively consider the interests and risks of both parties in the game, ensuring that the proportion of rewards and benefits shared by the reserves enterprises can not only motivate them to actively participate in reserves work, but also maintain the cooperation willingness of the NPOs. At the same time, it is necessary to flexibly adjust this ratio according to specific situations to adapt to changes in demand under different circumstances.

## 3 Numerical simulation

To further reveal the evolutionary paths and key driving factors of reserves strategies between NPOs and reserves enterprises, this study conducts numerical simulations using simulation software based on the theoretical analysis framework of evolutionary game models. Specifically focusing on the scenario dominated by the perfect competition, it emphasizes exploring the operational mechanism of government incentive policies on the evolutionary game system of emergency medical material production capacity reserves: among them, positive incentives exert an enabling effect by indirectly reducing the operating costs of both parties, while penalty measures inhibit speculative behaviors by compressing enterprises’ opportunity benefits, and the two jointly act on the equilibrium state of the reserves cost-benefit game. Through simulations, this section quantitatively analyzes the influence laws of key parameters such as government incentive intensity and penalty intensity on the evolutionary outcomes of the game system, providing solid data support and practical basis for the formulation of targeted policy recommendations in subsequent sections.

Taking the Chongqing region of China as the research context, this study selects the Chongqing Municipal Commission of Economy and Information Technology (as the government), the Chongqing Red Cross Society (as the NPO), and Chongqing Pharmaceutical (Group) Co., Ltd. (as the medical material reserves enterprise) as the practical data support for numerical simulations [23,39,40]. Given the complex interactive relationships among the above three parties and the difficulty in accurately quantifying the core parameters of their actual operations through direct measurement, this study does not set customized values based on specific scenarios. However, to ensure the universality and practical applicability of the evolutionary laws derived from the research, the numerical settings strictly adhere to the following three principles: (1) The numerical differences among variables are consistent with the actual conditions in the field of emergency medical material reserves; (2) All numerical settings align with the basic assumptions presented in the theoretical analysis section of this paper; (3) The fluctuation range of the initial values of variables is controlled within a reasonable interval to avoid imbalances in the initial state of the system, laying a stable foundation for subsequent parameter sensitivity analysis. Since the core objective of this study is to explore the evolutionary trends and inherent variation laws of the game system, setting values in accordance with the above principles can effectively achieve this goal. Based on this, the initial parameters of the model are set as follows: $\pi_{0}=0.1$, *R* = 10, $\kappa_{1}=0.9$, $\kappa_{2}=1$, *h* = 1, *K* = 0.5, *C*_1_ = 7, *C*_2_ = 8,*N*_1_ = 2, *N*_2_ = 3, *A*_1_ = 4, *A*_2_ = 2, ν=0.5, *B*_1_ = 10, *B*_2_ = 3, *B*_3_ = 4, $\chi_{1}=0.2$, $\chi_{2}=0.3$, $\chi_{3}=0.5$.

### 3.1 The impact of the ratio of reserves costs to reserves benefits on the evolutionary results

In order to achieve the reserves of emergency medical supplies production capacity, the establishment of cooperative relationships is particularly important. The construction of this relationship between the NPOs and the reserves enterprises are based on a balance of interests between both parties, which means that neither party can suffer losses in the cooperation. Therefore, the benefits and costs of both parties become key factors in strategy selection. According to the previous analysis, when the NPOs cooperates with the reserves enterprises, if the investment cost, reputation benefits, and social disaster reduction benefits can be reduced, it will greatly increase the possibility of the game system developing towards the direction of entrusted reserves.

**Fig 5:**
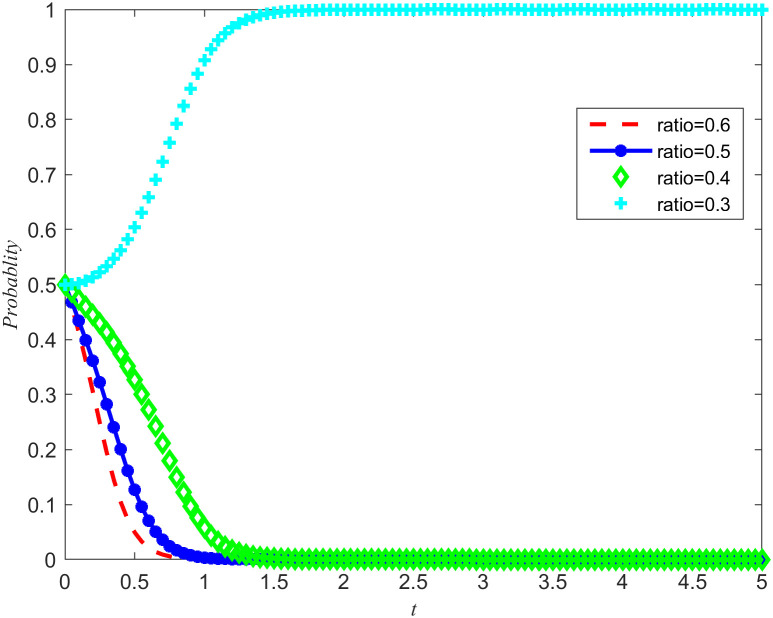
System evolution trajectory under different proportions of cost and benefit of the NPOs.

**Fig 6:**
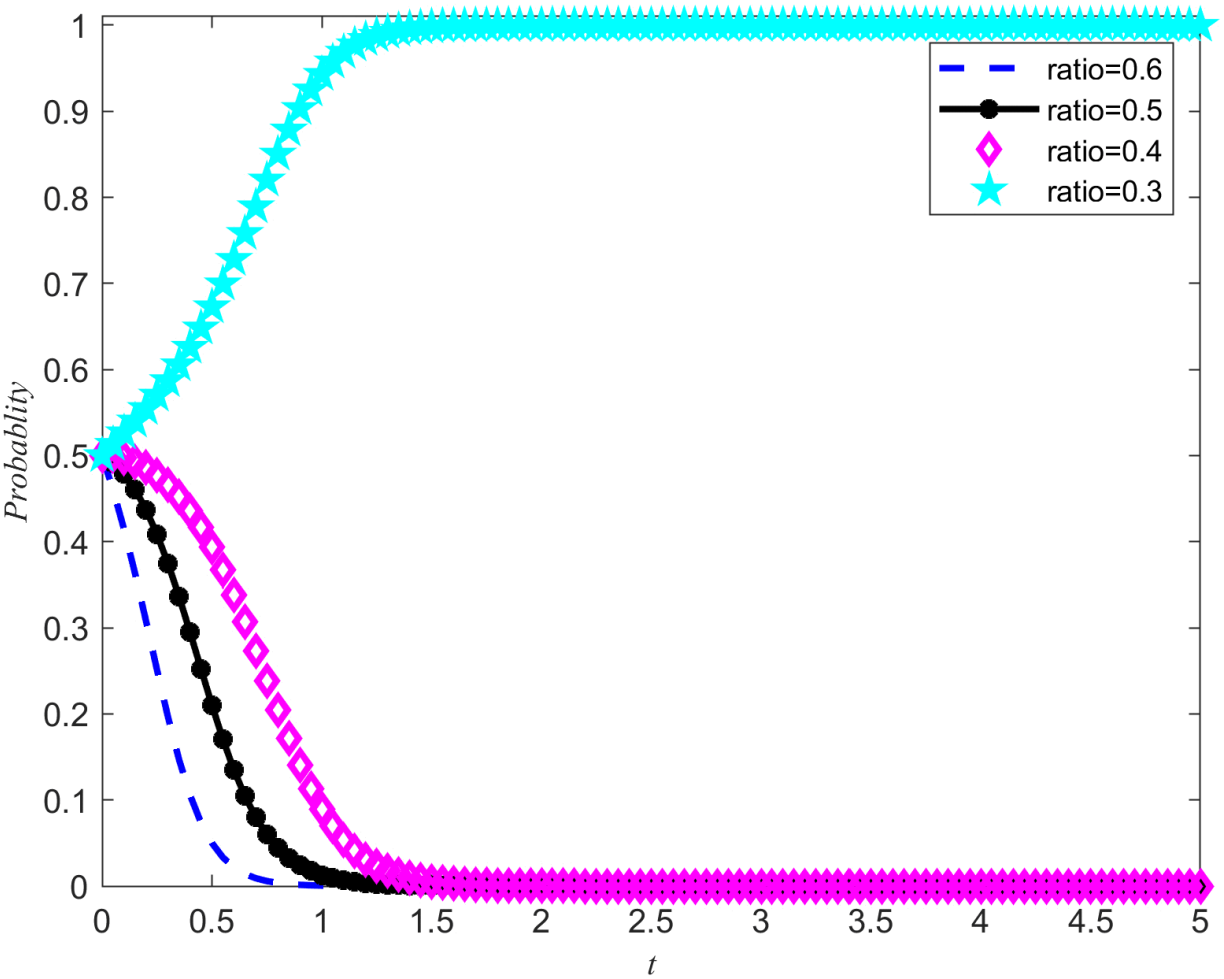
System evolution trajectory under different proportions of cost and benefit of the reserves enterprises.

In order to further explore the intrinsic relationship between costs and benefits, and to investigate the specific impact of perfect competition on the evolution of the emergency medical material production capacity reserves game system, a hypothetical scenario is set: while maintaining the stability of social disaster reduction benefits and reputation benefits, the ratio of reserves costs to benefits is adjusted to 0.3, 0.4, 0.5, and 0.6, respectively. Based on these parameter settings, a simulation of the game system was conducted, and the results are shown in Figures 5 and 6.

When the ratio of cost to benefit cannot promote the evolution of the direction of emergency medical material production capacity reserves between the NPOs and the reserves enterprises, goverment incentive policies will reduce the cost of emergency medical material production capacity reserves. To further explore the impact of goverment emergency reserves incentive policies on the evolutionary trajectory, when the ratio of cost to benefit is 0.6, the effect of cost subsidy intensity on the evolutionary outcome of the game system is observed, and the evolutionary result is Non-entrusted reserves, no reserves.

**Fig 7:**
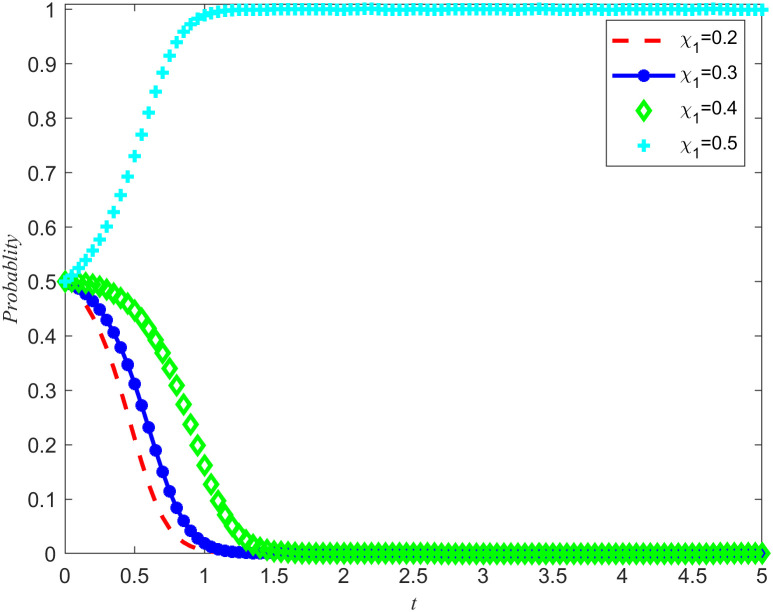
System Evolution Trajectory under Different Cost Subsidy Levels of the NPOs.

**Fig 8:**
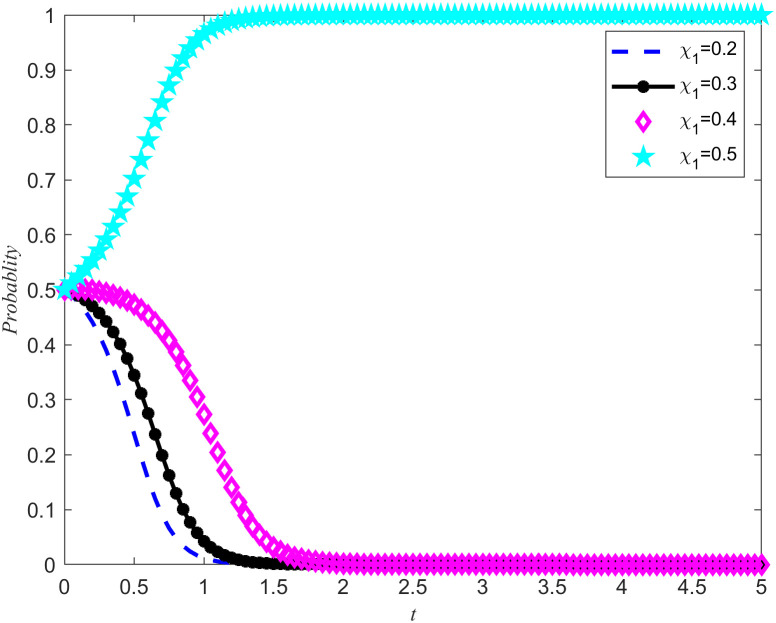
System evolution trajectory under different cost subsidy levels of the reserves enterprises.

Figures 7 and 8 shows the evolution trajectory of the system under different cost subsidy levels. When the NPOs does not entrust, but the enterprises carries out critical emergency medical material reserves, the NPOs will provide cost subsidies to the reserves enterprises with a subsidy coefficient. For the convenience of numerical simulation, the cost subsidy coefficients are taken as 0.2, 0.3, 0.4, and 0.5, respectively. The results are shown in Figures 7 and 8. Observing Figures 7 and 8, it can be observed that when the cost-benefit ratio between the nonprofits organizations and the reserves enterprises are relatively high, making it difficult for both parties to spontaneously form reserves cooperation, the introduction of cost subsidies becomes a key factor. Moreover, this cost subsidy has a threshold between 0.4 and 0.5. If the cost subsidy given to the reserves enterprises fail to reach this threshold, the reserves enterprises will lack sufficient motivation to evolve towards emergency medical material production capacity reserves. This means that in order to effectively incentivize reserves enterprises to participate in emergency medical supplies reserves, it is necessary to ensure that the cost subsidies provided reach or exceed this critical threshold. Meanwhile, Proposition 7 is validated in Figures 7 and 8.

**Fig 9:**
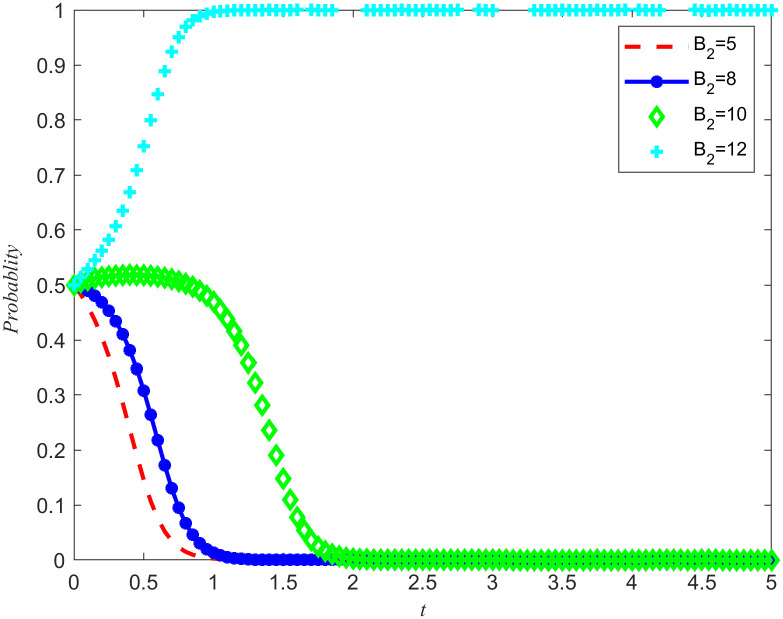
System Evolution trajectory under different reward levels for reserves achievements of the NPOs.

**Fig 10:**
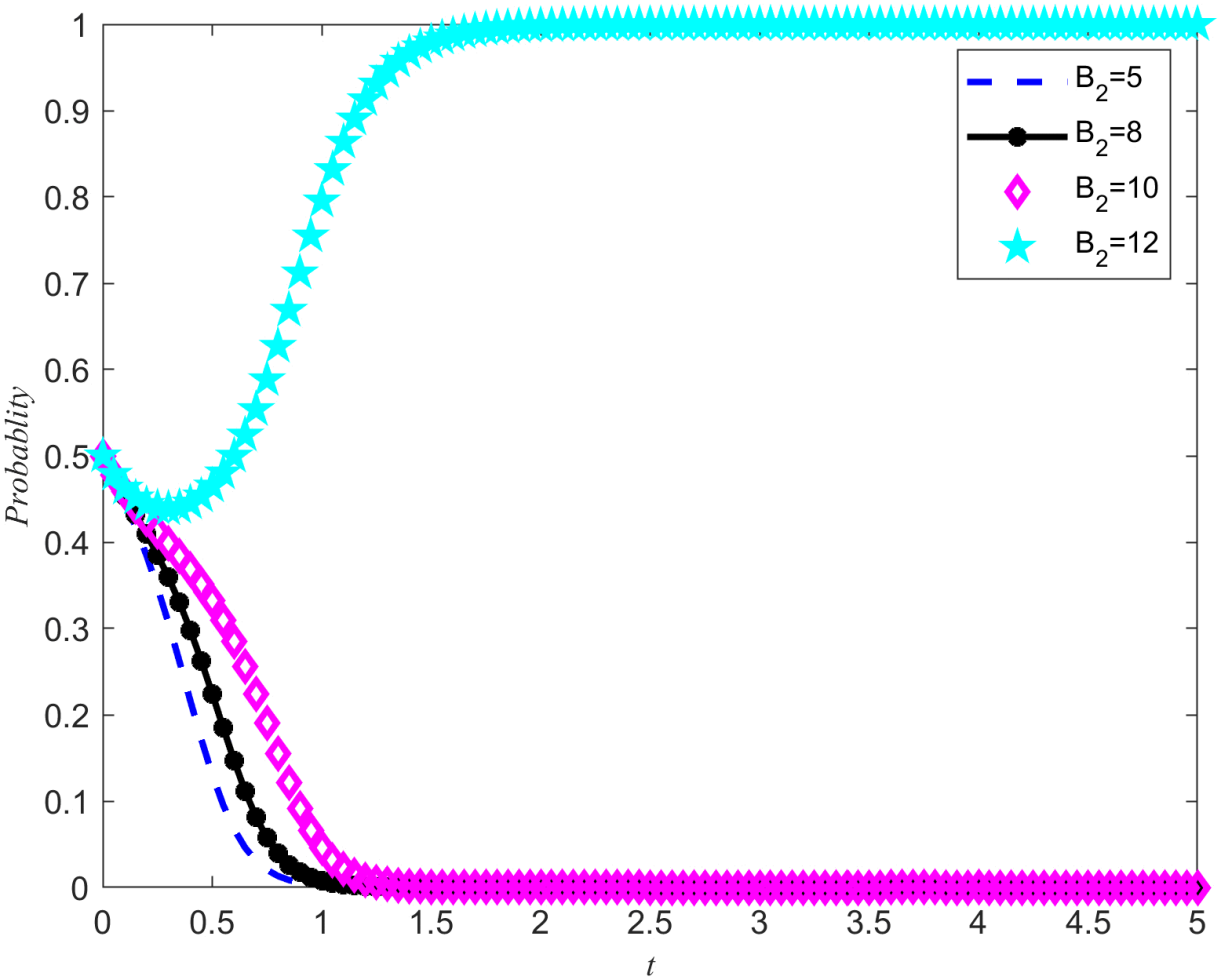
System evolution trajectory under different reward levels for reserves achievements of the reserves enterprises.

Observing the impact of reserves achievement rewards on evolutionary outcomes, Figs 9 and 10 show that reserves achievement rewards have a weaker initial incentive effect, while cost subsidies have a faster convergence speed on evolutionary outcomes, making their impact more direct. However, in the long run, the effect of achievement rewards is more significant, so a combination of two methods can be used to incentivize reserves enterprises. In addition, through the joint efforts of the NPOs and the reserves enterprises, the NPOs strengthens local construction, improves reserves planning and layout, and builds a reserves information platform. The reserves enterprises improve the production capacity and reserves level of emergency medical materials, which is expected to provide faster and high-quality medical material support in the event of public health incidents, generate more social benefits, and both parties can obtain more rewards for emergency reserves achievements, further ensuring the stability of the entrusted agency cooperation for emergency medical material production capacity reserves. Meanwhile, Proposition 7 is once again validated in Figs 9 and 10.

Based on the above simulations, both cost subsidies and rewards for reserves achievements can promote the evolution of the game system towards cooperation in emergency medical material production capacity reserves. When the NPOs simultaneously carry out reserves cost subsidies and reserves achievement rewards, the thresholds of both measures will be affected and changed simultaneously. In order to further investigate how cost subsidy intensity affects the evolutionary results of the game system, a specific reserves achievement reward value *B*_2_ = 5 was set, and four different cost subsidy intensities were selected, namely 0.2, 0.3, 0.4, and 0.5. By observing Figs 7–10 the evolution trajectory of the emergency medical supplies production capacity reserves game system under different cost subsidy levels can be obtained.

**Fig 11:**
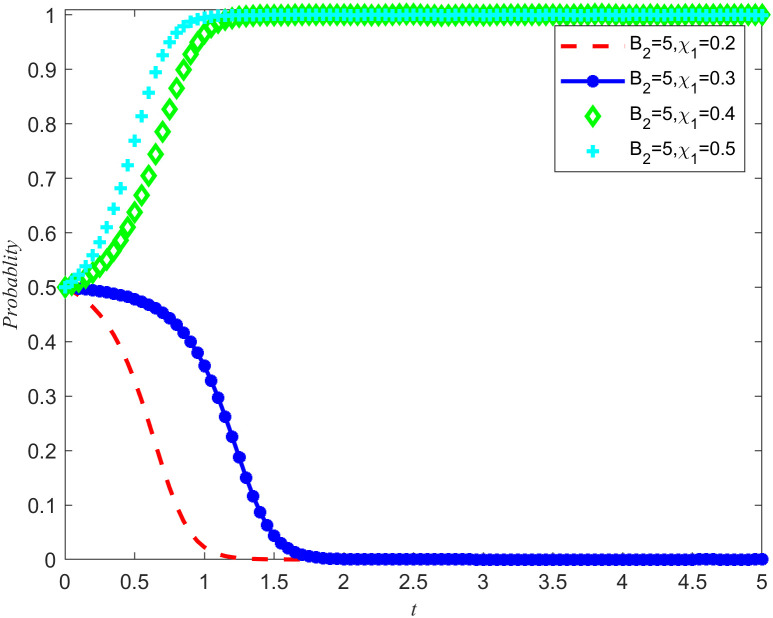
System evolution trajectory under different cost subsidy levels under reserves achievement rewards of NPOs.

**Fig 12:**
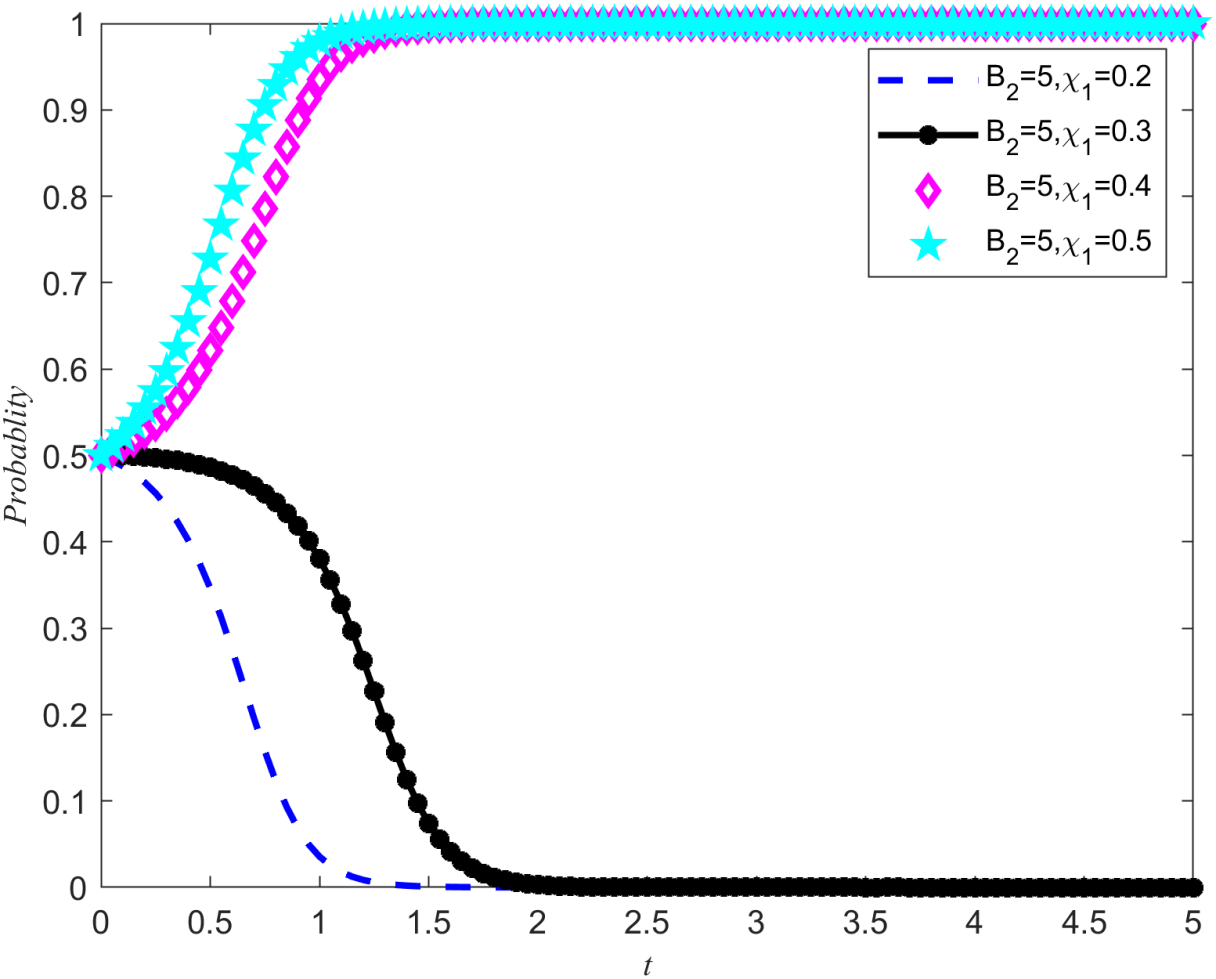
System evolution trajectory under different cost subsidy levels under reserves achievement rewards of the reserves enterprises.

If the rewards for the achievements of the reserves enterprises are reduced, it can cause a change in the cost subsidy threshold, and the two are positively correlated. By observing Figs 11 and 12, it can be seen that when the threshold for cost subsidies is lowered to between 0.3 and 0.4, the simultaneous use of achievement rewards and cost subsidy policies can promote the evolution of reserves enterprises toward reserves cooperation.

### 3.2 The impact of unilateral reserves willingness returns and reserves profits and losses on evolutionary outcomes

**Fig 13:**
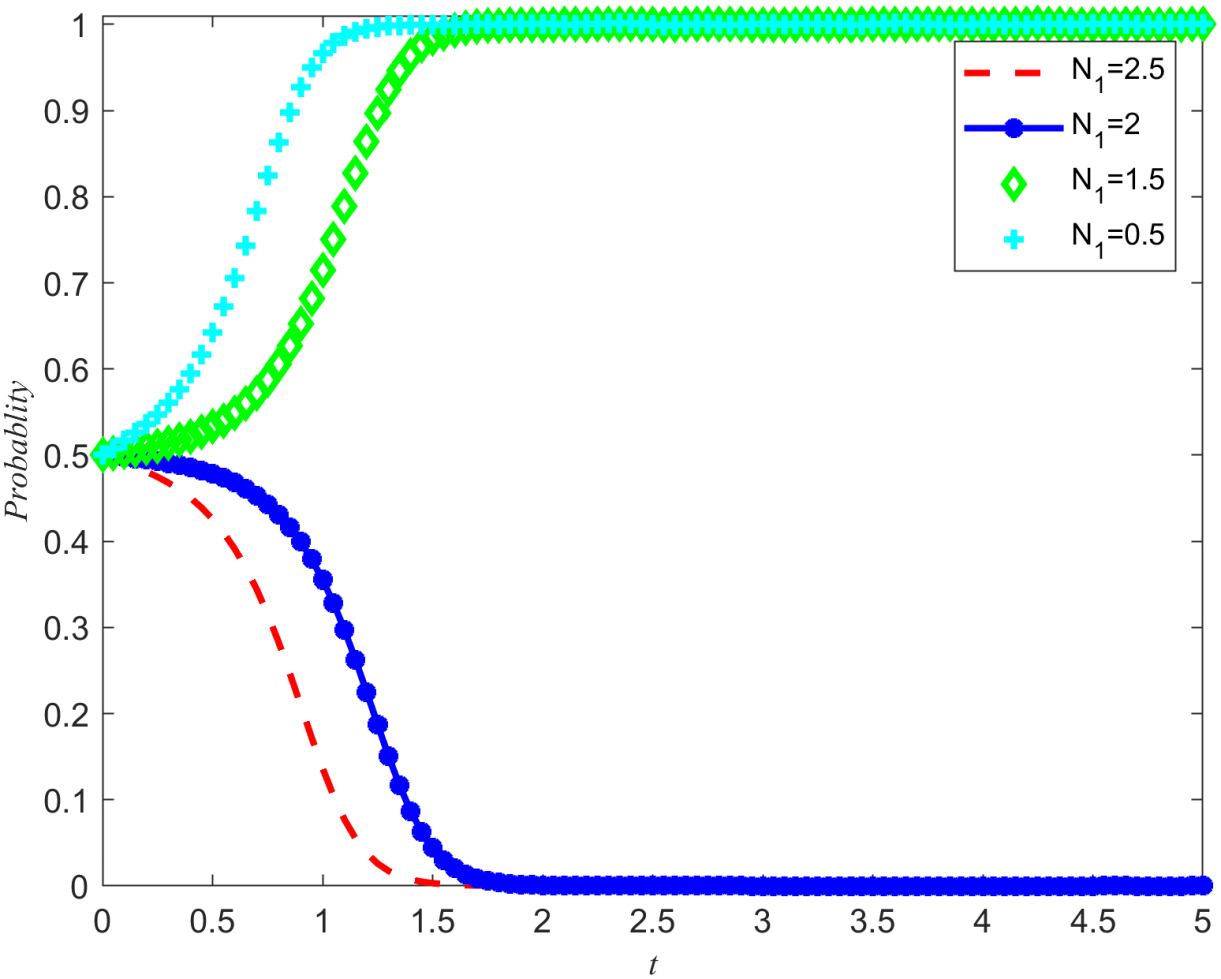
The impact of unilateral reserves intention returns on the evolutionary outcomes of the NPOs.

**Fig 14:**
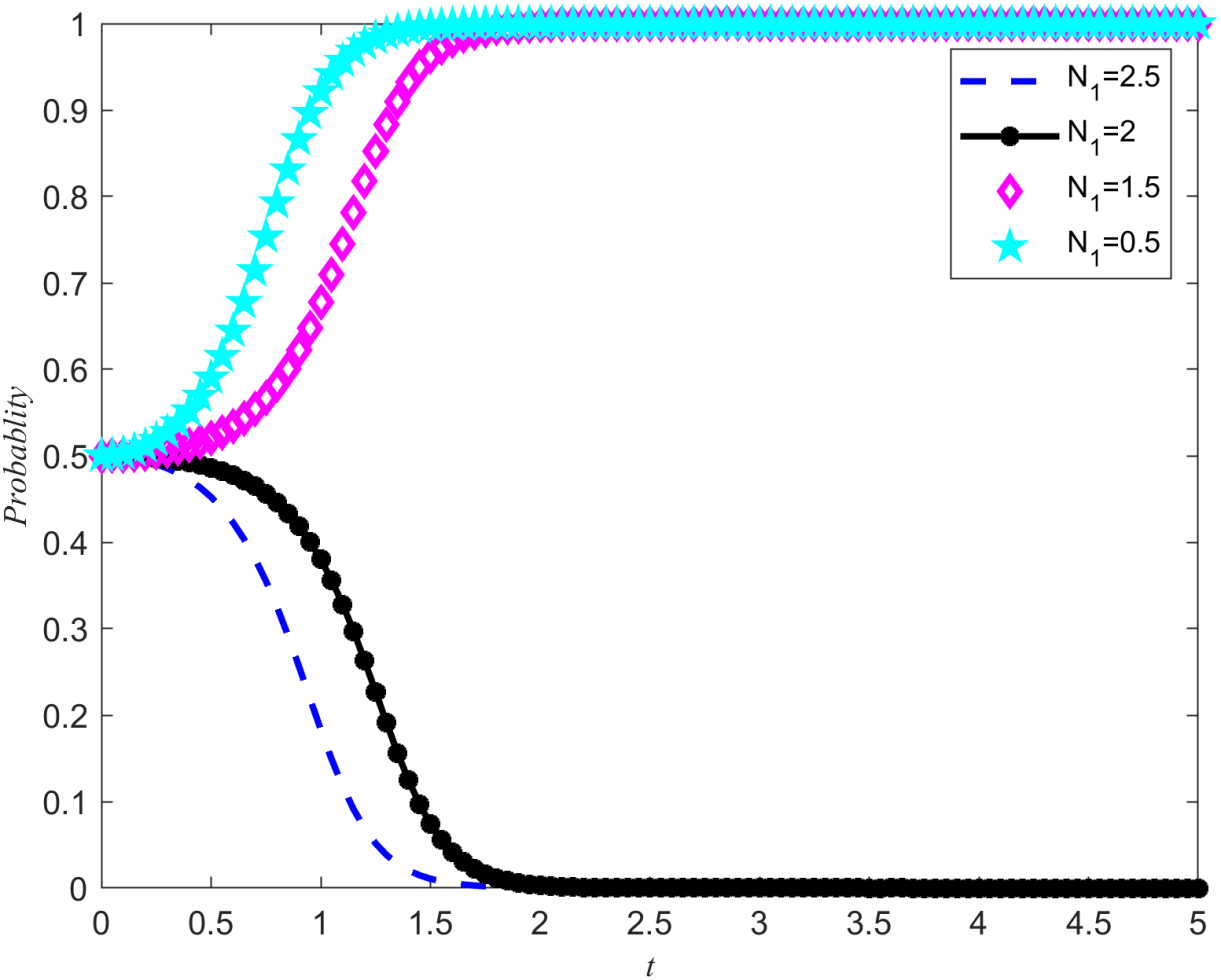
The impact of unilateral reserves intention returns on the evolutionary outcomes of the reserves enterprises.

**Fig 15:**
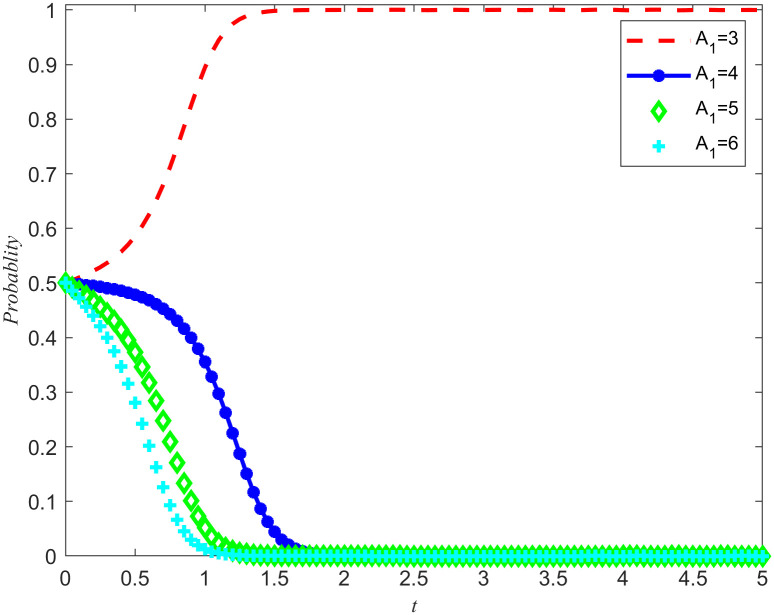
The impact of reserves profits and losses on the evolutionary outcomes of the NPOs.

**Fig 16:**
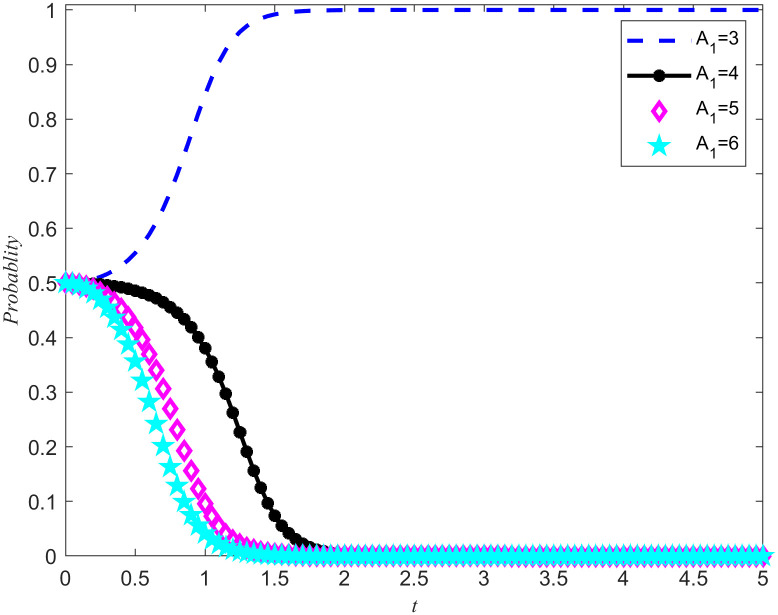
The impact of reserves profits and losses on the evolutionary outcomes of the reserves enterprises.

Take *B*_2_ = 5,$\chi_{2}=0.3$. From Figs 11 and 12, it can be seen that the enterprises choose rejecting reserves and the NPOs choose non-entrust reserves. In addition, the penalty base is set to 0, and the NPOs takes 0.5, 1.5, 2, and 2.5 from the unilateral reserves intention of the reserves enterprises, while keeping all other parameters unchanged. The simulation results are shown in Figs 13 and 14. In addition, assuming all other parameters remain unchanged, reserves cooperation costs of the NPOs are taken as 3, 4, 5, and 6, respectively, and the simulation results are shown in Figs 15 and 16.

Proposition 5 and Proposition 2 are validated in Figs 13–16. On the premise that the income of the NPOs and the reserves enterprises remain unchanged, observe the changes in unilateral reserves intention income and reserves cooperation costs, as well as the proportion of unilateral intention income and reserves cooperation costs to reserves income. By observing Figs 13–16, it can be observed that there are specific thresholds for both unilateral reserves willingness returns and reserves cooperation costs. It is worth noting that the threshold for unilateral reserves returns is actually lower than the threshold for reserves cooperation costs. This finding indicates that compared to unilateral reserves returns,reserves cooperation costs have a more significant impact on the ultimate strategic choices of NPOs and reserves enterprises. In other words, when considering whether to engage in reserves cooperation, both parties are more likely to be affected by reserves cooperation costs, rather than just focusing on the size of unilateral reserves returns. At the same time, the cooperation between the two parties in entrusting and acting as agents for the reserves of emergency medical supplies production capacity is conducive to improving their respective social reputation and sharing the benefits of social disaster reduction. At the same time, when the reserves reserves opportunity cost coefficients is high, it can effectively reduce the reserves cooperation costs and enhance the stability of the entrusted agency relationship for the reserves of emergency medical supplies production capacity.

**Fig 17:**
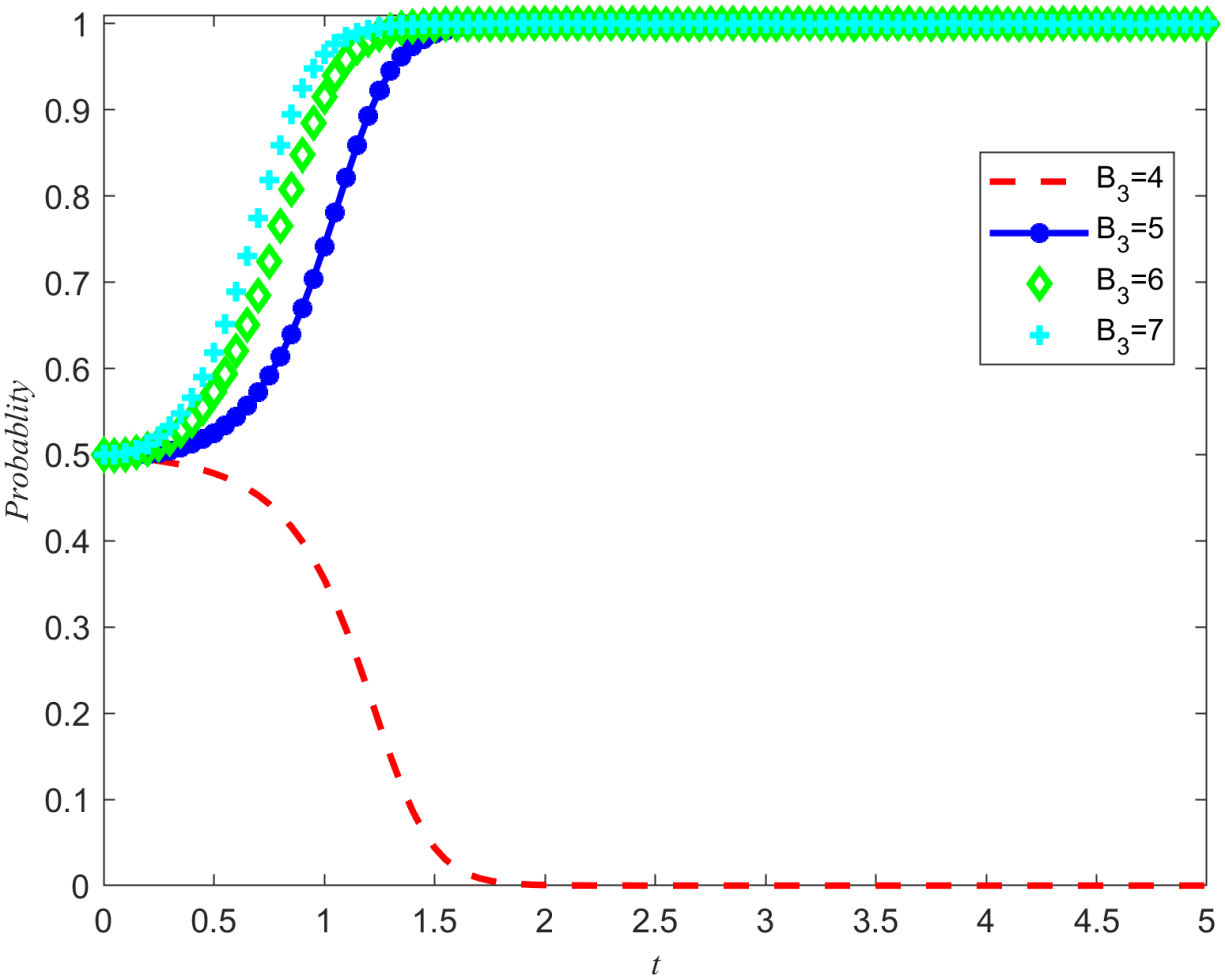
The impact of changes in punishment intensity on the evolutionary outcomes of the NPOs.

**Fig 18:**
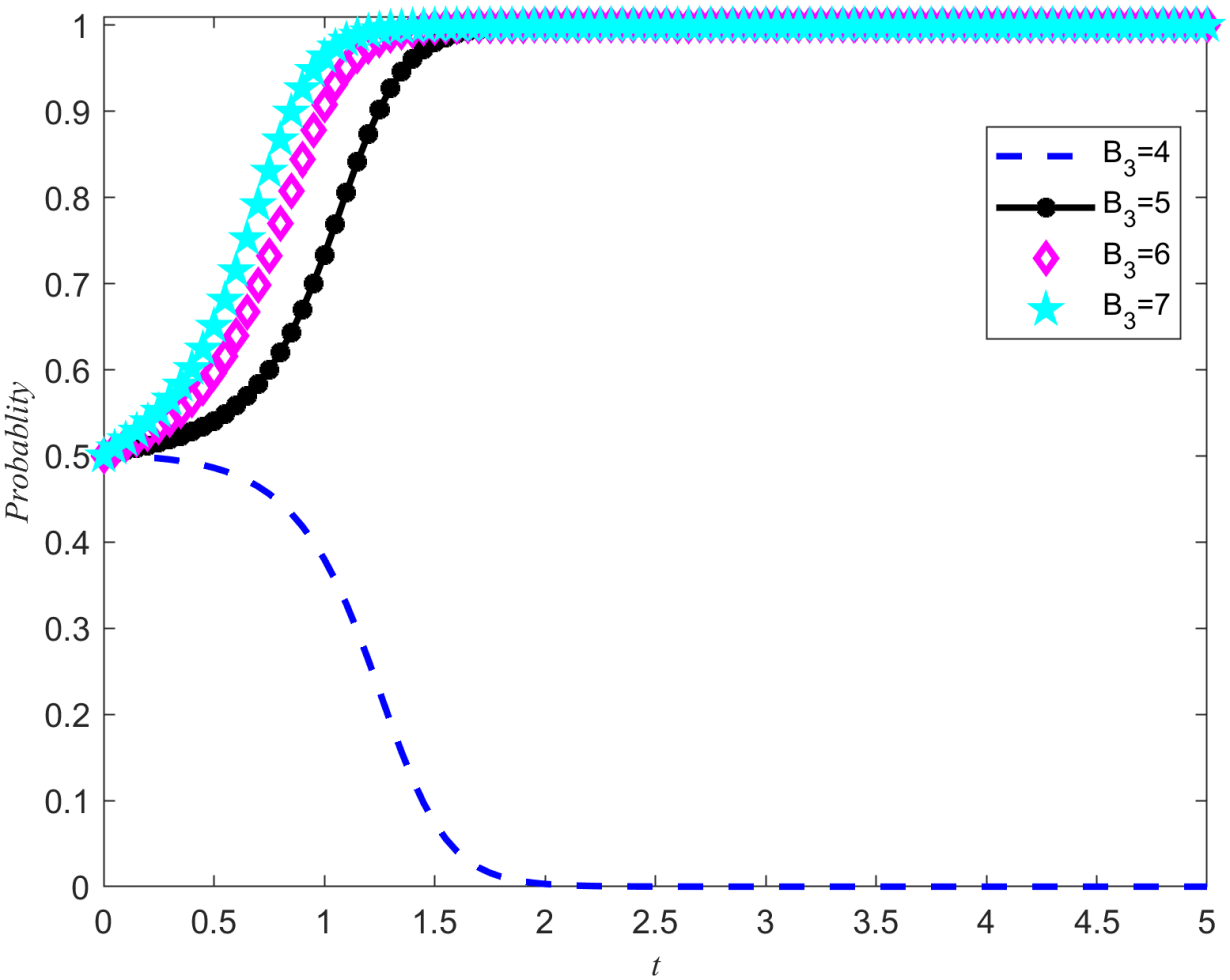
The impact of changes in punishment intensity on the evolutionary outcomes of the reserves enterprises.

At the same time, the introduction of punishment mechanisms has also played a positive role in improving the quality of reserves opportunity cost coefficients. Based on the simulation results of Figs 13–16, when *N*_2_ = 2 and *A*_1_ = 4, the system tends to evolve in a direction of Non-entrusted reserves or reserves. In order to further explore the impact of punishment on the game system, we set *N*_2_ = 2 while keeping other parameters constant, *A*_1_ = 4, And adjust the penalty base values to 4, 5, 6, and 7 respectively. Through this series of simulation experiments, we obtained the evolution results of the system under different penalty bases, as shown in Figs 17 and 18.

The simulation results in Figs 17 and 18 not only verify the accuracy of Proposition 8, but also further reveal the specific impact of punishment intensity on the system evolution results. When the punishment intensity is between 4 and 5, there is a clear threshold. This means that if the punishment is below this threshold and fails to exceed speculative returns, such punishment will not effectively guide the reserves enterprises to engage in fair competition. Only when the punishment exceeds this threshold will the system evolve towards the direction of developing emergency medical material production capacity reserves. In addition, with the increase of punishment intensity, the convergence speed of the game system also shows an accelerating trend. Although the macro level incentive policies for the cooperation of emergency medical supplies production capacity reserves have provided an opportunity for both sides to cooperate, there is still uncertainty in the implementation process. The punishment mechanism, as an effective means of constraint, can limit the speculative behavior of the reserves enterprises, thereby avoiding potential damage to the rights and interests of the NPOs. More importantly, the punishment mechanism plays a crucial role in driving both parties towards a stable strategy of entrusted reserves, providing strong guarantees for the realization of emergency medical material production capacity reserves.

## 4 Conclusions

Based on the core ideas and analytical methods of evolutionary game theory, this study constructs dynamic evolutionary game models for the cooperative production capacity reserves of emergency medical supplies between NPOs and reserves enterprises under two scenarios: “perfect competition” and “government incentive policy intervention.” Through theoretical deduction, the operational mechanisms of key factors—including one-time subsidies, revenue sharing, cost subsidies, and regulatory penalties—on the strategic choices of both parties are systematically analyzed. Furthermore, numerical simulation is used to quantitatively examine the impact of government incentive policies on the final decision-making equilibrium of the two types of subjects. The research results are summarized as follows:

(1) Under the synergistic effect of perfect competition and government incentive policies, multiple key factors—such as reputation value, reputation conversion capability, social disaster reduction benefits, and government incentive intensity—jointly drive the evolutionary game system of emergency medical supply production capacity reserves toward cooperative equilibrium. However, the impact of government incentive policies on the game system is not fixed: the achievement of reserves cooperation significantly depends on the reasonable setting of the incentive allocation coefficient. Excessive bias toward enterprises will weaken NPOs’ willingness to entrust reserves, while excessive inclination toward NPOs may reduce enterprises’ enthusiasm for reserves participation. Therefore, the government must carefully consider and scientifically calibrate this allocation coefficient when formulating relevant incentive policies.
(2) Government incentive policies are the key driving force for breaking through the threshold constraints of cooperative production capacity reserves and promoting the game system toward cooperative equilibrium. Their coordinated application with NPOs’ cost subsidies can significantly improve cooperation efficiency and stability through complementary advantages. Specifically, the reserves cost-benefit ratio is the core threshold variable determining cooperation: at low ratios, cooperative equilibrium can be easily achieved through the spontaneous operation of market mechanisms; once exceeding a specific threshold, perfect competition alone fails, and the game system tends to evolve toward non-cooperation. Government incentive policy intervention can effectively address this dilemma: on the one hand, compared with NPOs’ cost subsidies (which take effect quickly in the short term), government incentives exert long-term and fundamental effects, fundamentally lowering the cooperation threshold; on the other hand, the implementation of government incentives generates a linkage effect, significantly reducing the cost subsidy threshold for NPOs, further alleviating their participation pressure and enhancing their willingness to cooperate. Therefore, constructing a collaborative incentive model with government incentive policies as the core and NPOs’ cost subsidies as supplements can form a complementary effect of “long-term empowerment + short-term efficiency improvement,” efficiently breaking through the cost-benefit ratio threshold constraint and promoting the stable implementation and sustainable operation of cooperative production capacity reserves.
(3) Enterprises’ opportunity costs have a significant negative impact on the achievement of reserves cooperation, while opportunity benefits may drive the game system toward the “non-reserves” strategy. If the penalty intensity in government incentive policies is insufficient—i.e., lower than enterprises’ opportunity benefits—enterprises will most likely abandon reserves. In such cases, penalty intensity must be increased: only when penalties exceed a specific critical threshold can cooperative production capacity reserves be promoted. In addition, there is a positive correlation between the government’s penalty intensity and the cooperative performance of reserves enterprises: stricter penalties reduce enterprises’ opportunity benefits, strengthen their willingness to cooperate, and facilitate the achievement of emergency medical supply production capacity reserves goals.

Based on the above research conclusions and combined with practical implementation needs, to further improve the operability of the policy suggestions, this study refers to the relevant laws, regulations and policies on emergency medical supplies reserves in Chongqing, China (including relevant provisions on emergency supplies management, tax incentives, and reward and punishment mechanisms) to clarify various quantitative data and operational standards [23,39–44]. The detailed suggestions are as follows. It should be noted that the setting of various data involved in this paper is consistent with the current practical norms in Chongqing, which is for reference only. It can be flexibly adjusted and adapted according to the emergency management policies, economic development level and actual reserves needs of other regions, combined with the latest local policy orientation. At the same time, if the relevant policies are adjusted subsequently or there is room for supplement and improvement of the existing reference information, the data setting can be optimized accordingly.

(1) Optimize the Incentive Allocation Mechanism to Balance the Interest Demands of Multiple Subjects Focusing on “precision quantification and dynamic adaptation”, optimize the incentive allocation mechanism for emergency medical supplies production capacity reserves to ensure that the incentive policies are operable and verifiable. Clarify that the government’s emergency management department takes the lead and the financial department cooperates as the exclusive implementation subject of incentive allocation, responsible for the policy implementation and dynamic adjustment, which is consistent with the requirement of multi-department collaborative management specified in the “Measures for the Administration of Emergency Medical Supplies Reserves in Chongqing.” Refine the evaluation indicators for the contribution of multiple subjects: the efficiency of enterprises’ production capacity reserves is calculated by “actual reserves volume / promised reserves volume”, and the resource integration capacity of non-profit organizations (NPOs) is calculated by “coordinated supplies arrival time limit / agreed time limit.” Combined with the practical experience of emergency medical supplies reserves in Chongqing and relevant policy orientation, the incentive allocation coefficient interval is initially set at 0.4 to 0.6, and the basic allocation ratio between enterprises and NPOs can refer to 55% and 45% respectively. Specifically, it can be dynamically adjusted according to the actual contribution evaluation results of both parties to avoid excessive incentive bias towards a single subject. In the implementation of incentives, it is necessary to ensure a reasonable profit margin for enterprises and fully cover the entrusted coordination costs of NPOs, so as to simultaneously enhance the participation willingness of both parties. “The enterprise reserves participation rate increases by more than 10% and the NPOs’ willingness to undertake reserves increases by more than 15%” can be used as the reference for evaluating the implementation effect, which can be optimized and adjusted according to the actual implementation situation to promote the stable evolution of the game system towards cooperative equilibrium.
(2) Construct a “Government-led + NPO-supplemented” Collaborative Incentive System to Break Through Threshold Constraints Deepen the “government-led + NPO-supplemented” collaborative model to form a closed-loop complementary mechanism featuring “long-term empowerment + short-term efficiency improvement”, effectively reduce the cooperation threshold and improve cooperation efficiency, which is consistent with the emergency supplies reserves management principle of “hierarchical responsibility and collaborative guarantee” in Chongqing. At the government level, combined with the relevant requirements on tax reduction and exemption in the “Measures for the Administration of Tax Preferential Policies Enjoyed by Chongqing Commodity Reserves Management Companies and Their Directly Affiliated Warehouses” and the calculation of the operation costs of local reserves enterprises in Chongqing, the specific standards for tax incentives for reserves enterprises can refer to reducing enterprise income tax by 5% to 8% according to the reserves capacity, and the specific ratio can be adjusted according to the latest tax policies. Standardize the payment process of reserves compensation funds, and it is recommended to implement quarterly payment. For the delayed payment part, liquidated damages can be paid at a rate of 0.05% per day to protect the legitimate rights and interests of enterprises and NPOs, echoing the requirement of standardized management of reserves funds in the “Measures for the Administration of Emergency Medical Supplies Reserves in Chongqing.” At the NPOs level, give play to the advantages of flexibility and efficiency, and simplify the application process for short-term subsidies. After enterprises and relevant subjects submit the reserves service agreement and cost details, the review shall be completed within 3 working days, and the subsidy can be issued at 3% to 5% of the coordinated supplies amount to quickly respond to emergency reserves needs. Establish a monthly docking mechanism between the government and NPOs to achieve policy linkage. “The cooperation threshold is reduced by more than 20% and the emergency supplies response time limit is shortened by more than 15%” can be used as the reference for evaluating the implementation effect, which is in line with the work requirement of “efficient allocation and rapid guarantee” of relief supplies in Chongqing, so as to realize the complementary advantages of both parties and improve cooperation efficiency and stability.
(3) Improve the Penalty Constraint Mechanism to Strengthen the Rigidity of Policy Implementation Establish a hierarchical and classified penalty constraint mechanism to strengthen the rigidity of policy implementation, restrict the short-term speculative behaviors of subjects, and ensure the achievement of reserves goals, which is consistent with the relevant provisions on the accountability of enterprises undertaking reserves for violations of regulations specified in the “Measures for the Administration of Emergency Medical Supplies Reserves in Chongqing.” Construct a hierarchical penalty system directly linked to enterprises’ opportunity benefits, ensuring that the penalty intensity is not only higher than the potential opportunity benefits of enterprises but also exceeds the critical threshold, so as to reduce the attractiveness of the “non-reserves” strategy. Clarify the reference for hierarchical penalty gradients: if the opportunity benefit is not more than 100,000 yuan, the fine amount can refer to 1.2 times the opportunity benefit; if the opportunity benefit is more than 100,000 yuan and not more than 500,000 yuan, the fine amount can refer to 1.5 times the opportunity benefit; if the opportunity benefit is more than 500,000 yuan, in addition to the fine according to the corresponding ratio, consideration may be given to revoking the enterprise’s policy support qualification within 3 years and including it in the joint disciplinary list for untrustworthy entities, and the specific penalty standards can be adjusted according to the latest laws and policies. Strengthen regular supervision, which is recommended to be carried out by the government’s emergency management department together with a third-party institution, completing a comprehensive evaluation every six months, and notifying and criticizing the relevant responsible persons who fail to implement the penalty mechanism. “The enterprise default rate is reduced to less than 5% and the reserves goal completion rate reaches more than 95%” can be used as the reference for assessment, which is compatible with the physical reserves requirements in Chongqing, forming a closed-loop management of “adequate incentives and strong penalties” to ensure the effective implementation of various reserves policies.

## 5 Limitations and future work

This study employs surrogate indicators and indirect data to conduct quantitative analysis, which has certain limitations. Future research can further optimize the quantitative models of these two aspects by conducting special surveys, obtaining microdata reserved from government-enterprise cooperation, and introducing questionnaire surveys and in-depth interviews, thereby enhancing the accuracy of research conclusions.

## Supporting information

S1 File SourceCode. SourceCodes for simulations. (SourceCode.zip) Contains two files: Sourcecode1 and Sourcecode2.
(ZIP)
